# Extracellular sialyltransferase st6gal1 in breast tumor cell growth and invasiveness

**DOI:** 10.1038/s41417-022-00485-y

**Published:** 2022-06-08

**Authors:** Nitai C. Hait, Aparna Maiti, Rongrong Wu, Valerie L. Andersen, Chang-Chieh Hsu, Yun Wu, Digantkumar G. Chapla, Kazuaki Takabe, Michael E. Rusiniak, Wiam Bshara, Jianmin Zhang, Kelley W. Moremen, Joseph T. Y. Lau

**Affiliations:** 1 Department of Surgical Oncology, Roswell Park Comprehensive Cancer Center, Elm & Carlton Streets, Buffalo, NY 14263 USA; 2 Department of Molecular & Cellular Biology, Roswell Park Comprehensive Cancer Center, Elm & Carlton Streets, Buffalo, NY 14263 USA; 3 Department of Biomedical Engineering, University at Buffalo, The State University of New York, Buffalo, NY 14260 USA; 4 Complex Carbohydrate Research Center, University of Georgia, 315 Riverbend Road, Athens, GA 30602 USA; 5 Department of Biochemistry and Molecular Biology, University of Georgia, 315 Riverbend Road, Athens, GA 30602 USA; 6 Department of Pathology, Roswell Park Comprehensive Cancer Center, Elm and Carlton Streets, Buffalo, NY 14263 USA; 7 Department of Cancer Genetics & Genomics, Roswell Park Comprehensive Cancer Center, Elm and Carlton Streets, Buffalo, NY 14203 USA

**Keywords:** Breast cancer, Cell biology

## Abstract

The sialyltransferase ST6GAL1 that adds α2–6 linked sialic acids to N-glycans of cell surface and secreted glycoproteins is prominently associated with many human cancers. Tumor-native ST6GAL1 promotes tumor cell behaviors such as invasion and resistance to cell stress and chemo- and radio-treatments. Canonically, ST6GAL1 resides in the intracellular secretory apparatus and glycosylates nascent glycoproteins in biosynthetic transit. However, ST6GAL1 is also released into the extracellular milieu and extracellularly remodels cell surface and secreted glycans. The impact of this non-canonical extrinsic mechanism of ST6GAL1 on tumor cell pathobiology is not known. We hypothesize that ST6GAL1 action is the combined effect of natively expressed sialyltransferase acting cell-autonomously within the ER-Golgi complex and sialyltransferase from extracellular origins acting extrinsically to remodel cell-surface glycans. We found that shRNA knockdown of intrinsic ST6GAL1 expression resulted in decreased ST6GAL1 cargo in the exosome-like vesicles as well as decreased breast tumor cell growth and invasive behavior in 3D in vitro cultures. Extracellular ST6GAL1, present in cancer exosomes or the freely soluble recombinant sialyltransferase, compensates for insufficient intrinsic ST6GAL1 by boosting cancer cell proliferation and increasing invasiveness. Moreover, we present evidence supporting the existence novel but yet uncharacterized cofactors in the exosome-like particles that potently amplify extrinsic ST6GAL1 action, highlighting a previously unknown mechanism linking this enzyme and cancer pathobiology. Our data indicate that extracellular ST6GAL1 from remote sources can compensate for cellular ST6GAL1-mediated aggressive tumor cell proliferation and invasive behavior and has great clinical potential for extracellular ST6GAL1 as these molecules are in the extracellular space should be easily accessible targets.

## Introduction

The sialyltransferase ST6GAL1, mediating the α2,6-sialylation of N-glycans, is prominently associated with human cancers, including ovarian, prostate, pancreatic, colon, lung, colorectal, pancreatic, gastric, breast cancers, and acute myeloid leukemia [1–3]. Elevated cancer ST6GAL1 is often correlated with high tumor grade, metastasis, and poorer patient prognosis [3–7]. Furthermore, ST6GAL1-mediated α2,6-linked sialylation has been implicated in activating PI3K/AKT, epidermal growth factor receptor (EGFR) in some cancers, and cell growth and proliferation [3, 8]. In addition, ST6GAL1-mediated sialylation modulates cell surface receptor function, including integrin, death receptor, TNFR1, and the promotion of epithelial to mesenchymal transition (EMT) and resistance to chemo- and radio- treatments [1, 3, 9, 10]. These and related studies have driven the idea that ST6GAL1 is a cancer-promoting factor, including breast cancer [11]. However, a few reports correlated better long-term patient survival among individuals with the most aggressive cancers and the highest cancer ST6GAL1 [12, 13]. This conundrum brings to question whether cancer ST6GAL1 overexpression is beneficial or ultimately detrimental to long-term patient outcomes and highlights the incomplete mechanistic understanding of how ST6GAL1 is involved in cancer progression. Canonically, functioning ST6GAL1 resides within the intracellular ER-Golgi secretory complex, where sialylation of nascent cell surface and secreted components occurs cell-autonomously. It is within the cell-autonomous context that ST6GAL1 involvement in cancer progression has been interpreted.

Aggressive breast cancer is a highly heterogeneous disease caused by a variety of distinct cell-intrinsic genetic alterations in mammary epithelial cells, leading to vastly heterogenic disease manifestation in individual patients and predominantly affecting patient prognosis and treatment options [14]. Cancer cell-extrinsic mechanisms, which are poorly understood, are also believed to contribute to disease progression and the heterogeneous genetic mutations with diverse presentations. Elevated N-glycan signatures are present in the disseminated cancer cells in breast cancer patients [15], and heterogeneity in α2,6-linked sialic acids have been suggested to potentiate the invasion of aggressive breast cancer cells [11].

In addition to the canonical intracellular ER-Golgi locale, catalytically active ST6GAL1 is also present in the extracellular spaces and systemic circulation. The physiologic roles of these extracellular glycosyltransferases have received scant attention, but we and others have identified extracellular ST6GAL1 as a potent modifier of hematopoiesis, inflammatory cell production, B cell differentiation, and proliferation, and in the sialylation of the anti-inflammatory IgG [16–22]. Furthermore, ST6GAL1 is released into the extracellular milieu from cancer cells and healthy non-involved sources such as the liver [18, 23]. We hypothesize that ST6GAL1 action is the combined contribution of intracellular cell-native ST6GAL1 acting cell-autonomously and the extracellular ST6GAL1 acting non-cell-autonomously.

We confirm that cancer ST6GAL1 is variably expressed in triple-negative breast cancer (TNBC) clinical specimens that confer heterogeneous presence of α2,6-sialic acids on aggressive breast tumors. Breast cancer cell-native ST6GAL1 is associated with metastatic progression of aggressive breast cancer, based on online KM-plotter analysis, but we also observed that high expression of cancer ST6GAL1 mRNAs is associated with a favorable relapse-free survival (RFS) in TNBC patients. Cancer cell-native ST6GAL1 is preferentially released in association with exosome-like particles and smaller exomere-like particles. Exosome-like particles from ST6GAL1-high cancer cells elicit increased cell proliferation and invasiveness in cancer cells with less native ST6GAL1 expression. Our data further suggest the presence of undefined cofactors within the cancer exosomes, and these cofactors potentiate the action of extracellular ST6GAL1. Extracellular ST6GAL1 and the undefined exosome cofactors can compensate cell-intrinsic ST6GAL1 expression and potentiate aggressive cancer cell growth and proliferation, cancer cell invasion, EMT, CSCs (cancer stem cells) transcription factors, and serum-withdrawal stress-mediated cancer cell apoptosis. Our data highlights a previously unappreciated, non-cell-autonomous mechanism linking extracellular ST6GAL1 in cancer pathobiology.

## Materials and methods

### Cells and reagents

MCF7, MDA-MB-231 (MDA-231), BT-474, T-47D, ZR-75-1, E0771, and BT-549 cells were obtained from ATCC, Virginia, USA. LM-2–4 cells were kindly provided by Dr. John Ebos, Roswell Park Comprehensive Cancer Center (RPCCC)(NY, USA). 4T1 parental and metastatic clone 4T1.2 cells were donated by Prof. Cheryl L. Jorcyk, Boise State University (ID, USA). Breast cancer brain metastatic clone MDA-231/BR was obtained from Dr. Patricia S. Steeg, National Cancer Institute, Bethesda, MD, USA. MCF7 cells were cultured in phenol red-free improved minimum essential medium supplemented with 0.25% glucose and 10% fetal bovine serum (FBS). BT-474, BT-549, 4T1, 4T1.2, and E0771 cells were grown in RPMI medium with 10 % FBS. T-47D, ZR-75-1, MDA-231, LM-2–4, and MDA-231/BR cells were grown in DMEM medium with 10% FBS. All cell culture media were supplemented with penicillin (100 units/ml) and streptomycin (100 µg/ml). All experiments were performed during the logarithmic growth phase, and cell lines were passaged for no more than 6 weeks. All cell lines used here are certified in our laboratory to be mycoplasma-free. Cells were plated 24 h before treatment. Recombinant rat secretory ST6GAL1 enzyme (rST6G) [24] was produced by Dr. Kelley W. Moremen, University of Georgia, Georgia, USA. CMP-sialic acid (CMP-Sia)(MilliporeSigma, Massachusetts, USA), DAPI (BioLegend, CA, USA; 0.5 µg/ml), Crystal Violet (MilliporeSigma, Massachusetts, USA), Matrigel (Biosciences, CA, USA), and Cell Counting Kit-8 (CCK-8), WST-8 was from Dojindo Molecular Technologies, MD, USA. shRNA targeting mouse ST6GAL1 (shST6GAL1 #1, established and validated in Lau Laboratory; shST6GAL1 #2, Sigma, cat # TRCN0000018819, sequence CCGGCGAGAGATTGATAATCATGATCTCGAGATCATGATTATCAATCTCTCGTTTTT; shST6GAL1 #3, Sigma, cat # TRCN0000018821, sequence CCGGCCAGATCTGATTCAGCCGAATCTCGAGATTCGGCTGAATCAGATCTGGTTTTT), human ST6GAL1 (shST6GAL1 #1, Sigma, cat # TRCN0000035432, sequence CCGGCGTGTGCTACTACTACCAGAACTCGAGTTCTGGTAGTAGTAGCACACGTTTTTG; shST6Gal1 #2, Sigma, cat # TRCN0000035429, sequence CCGGCGCTGCTCTATGAGAAGAATTCTCGAGAATTCTTCTCATAGAGCAGCGTTTTTG), and shControl (pLKO.1) were used to knockdown ST6GAL1. Lipofectamine 3000 transfection reagent (Invitrogen, MA, USA) was used for the transfection of shRNA plasmids. Successful knockdown was confirmed by immunoblotting using anti-ST6GALI goat polyclonal antibody (R&D Systems, MN, USA; cat. # AF5924) and qPCR analysis.

### Immunoblotting

Cell lysates were used for Western blot analysis as previously described [25]. Briefly, cells were washed twice with the ice-cold 1X PBS and lysed by probe-sonication in the lysis buffer [20 mM Tris (pH 7.4), 20% glycerol, 150 mM NaCl, 0.5% NP-40, 5 mM sodium orthovanadate, 40 mM β-glycerophosphate, 15 mM NaF, 0.5 mM 4-deoxypyridoxine, and protease inhibitor cocktail (Sigma)]. Unbroken cells were removed by centrifugation at 700 × g for 10 min at 4 °C. Protein concentrations were measured by the Bradford method (Bio-Rad, CA, USA). A total of 25 μg of protein was loaded per lane and separated by 10% SDS-PAGE and transferred to nitrocellulose membranes. Membranes were blocked with 5% blocking solution in 1X TBST buffer (both from Bio-Rad) at room temperature for 1 h. Membranes were incubated overnight at 4 °C with the following primary antibodies (1:1,000 dilution) in TBST buffer (Bio-Rad). Membranes were washed thoroughly with TBST buffer at room temperature and followed by incubation (1 h at room temperature) with the rabbit or mouse, or goat secondary antibodies (1: 5,000 in TBST with 1% blocking solution). Membranes were washed thoroughly at room temperature and developed with the enhanced chemiluminescence (ECL) western blotting substrate (#32106; Pierce/Thermo Fisher Scientific, MA, USA). Western blot images were obtained by exposing the membranes to X-ray films (Thermo Fisher Scientific, MA, USA).

The following primary antibodies were used for western blot analysis: β-tubulin, Cytochrome c, Caspase 3 cleaved product, PARP, N-cadherin, E-cadherin, Snail are from Cell Signaling Technology, MA, USA; TSG101, CD63, and GM130 are from Santa Cruz Biotechnology, CA, USA. In addition, the following secondary antibodies were used for Western blot analysis: peroxidase-conjugated affipure goat anti-rabbit IgG (#111-035-045) and goat anti-mouse IgG (#115-035-062) (both from Jackson ImmunoResearch Laboratories, Inc., PA, USA), and anti-Goat IgG HRP-conjugated antibody (#HAF109, R&D Systems, Inc. MN, USA).

### In vitro sialyltransferase assay

Sialyltransferase assays using cell extracts were carried out as described previously [18]. Briefly, 1.4 µl of cell lysates were mixed with Acceptor A (Galβ1,4GlcNAc-O-Bn; Toronto Research Chemicals Inc, Ontario, Canada) for ST6GAL1 activity or Acceptor B (Galβ1,3GlcNAc-O-Bn; Toronto Research Chemicals Inc, Ontario, Canada) for ST3GAL6 enzyme activity, the substrate CMP-Sia^H3^ in the assay buffer (50 mM sodium cacodylate pH 6.5, 0.5% Triton X-100). The enzyme reaction was performed at 37 °C for 1 h. After the enzymatic reaction, the enzyme product (Sia^H3^-Acceptor) was separated by using a Waters Sep-Pak® C18 column cartridge. Liquid scintillation counting of the methanol flow-through counts radioactivity from reacted product exclusively. Enzyme activity was calculated as fmol product/min/mg of cell extract protein.

### Real-time PCR

Quantitative real-time PCR (qPCR) analysis was performed as described before [25]. Briefly, total RNA was prepared with Trizol (Life Technologies, CA, USA). DNA contamination was removed from RNA with DNase treatment (Qiagen, MD, USA). RNA (2 μg) was reverse transcribed with the high-capacity cDNA Archive kit (Life Technologies). cDNAs were diluted 10-fold (target genes) and 100-fold (house-keeping gene Glyceraldehyde 3-phosphate dehydrogenase (GAPDH), and amplified with SYBR Green qPCR on CFX96 cycler (Bio-Rad). Gene expression levels were calculated by the 2^−(ΔΔCT)^ method [26], and for some experiments, 2^−(ΔCT)^ method [27] were normalized to GAPDH expression. Following real-time PCR, primers are used in this manuscript.

Human-ST6GAL1-F: 5′-GCAAAGATCAGAGTGAAACAG-3′, human-ST6GAL1-R: 5′-CACCTCATCGCAGACATG-3′; mouse-ST6GAL1-F: 5′-CTTGGCCTCCAGACCTAGTAAAGT-3′, mouse-ST6GAL1-R: 5′-TCCCTTTCTTCCACACGCAGATGA-3′; human-GAPDH-F: 5′- CTTAGCACCCCTGGCCAAG, human-GAPDH-R: 5′-GATGTTCTGGAGAGCCCCG-3′; mouse-GAPDH-F: 5′-TCACCATCTTCCAGGAGGGA-3′, mouse-GAPDH-R: 5″- GCATTGCTGACAATCTTGAGTGAG-3′; human-Twist1-F: 5′-CTCAAGAGGTCGTGCCAATC, human-Twist1-R: 5′-CCCAGTATTTTTATTTCTAAAGGTGTT-3′; human-Slug-F: 5′- CGAACTGGACACACATACAGTG-3′, human-Slug-R: 5′-CTGAGGATCTCTGGTTGTGGT-3′; human-Aldh1-F: 5″- AACTCCTCTCACTGCTCTCCACG-3′, human-Aldh1-R: 5′- GTCACCCTCTTCAGATTGCTTTTCC-3′; human-c-Myc-F: 5′- CACGAAACTTTGCCCATAGC-3′, human-cMyc-R: 5′-GCAAGGAGAGCCTTTCAGAG-3′; human-Nanog-F: 5′-TTT GTG GGC CTGAAG AAA ACT-3′, human-Nanog-R: 5′-AGG GCT GTC CTG AAT AAG CAG-3′; human-Oct4-F: 5′- CCTGAAGCAGAAGAGGATCA-3′, human-Oct4-R: 5′-CCGCAGCTTACACATGTTCT-3′; human-E-Cadherin-F: 5″- CGTGAGCATCCAGGCAGTGGTAGC-3′, human-E-Cadherin-R: 5′- GAGCCGCCGCCGCAGGAAG-3′; human-N-Cadherin-F: 5′-TCAGGCGTCTGTAGAGGCTT-3′, human-N-Cadherin-R: 5′-ATGCACATCCTTCGATAAGACTG-3′; human-Snail-F: 5′-GGCAATTTAACAATGTCTGAAAAGG-3′, human-Snail-R: 5′-GAATAGTTCTGGGAGACACATCG-3′; human-CD44-F: 5′-AGAAGGTGTGGGCAGAAGAA-3′, human-CD44-R: 5′-AAATGCACCATTTCCTGAGA-3′; human- Vimentin-F: 5’AGTCCACTGAGTACCGGAGAC-3′, human- Vimentin-R: 5′- CATTTCACGCATCTGGCGTTC-3″; human-Sox2-F: 5′-GAGCTTTGCAGGAAGTTTGC-3′, human-Sox2-R: 5′-GCAAGAAGCCTCTCCTTGAA-3′.

### Isolation of extracellular vesicles (EVs) by ultracentrifugation

As previously described, EVs were isolated from the cancer cell-conditioned medium with minor modifications [23, 28]. Briefly, confluent breast cancer cells were cultured in serum-free media for 48 h. The supernatants were collected and centrifuged at 500 × *g* for 20 min at 4 °C to discard cells. The supernatants were further centrifuged at 10,000 × g for 30 min at 4 °C to discard cell debris and apoptotic bodies. Finally, the supernatants were ultra-centrifuged at 100,000 × g for 90 min at 4 °C to precipitate the EVs. The EVs were resuspended with 20 volumes of ice-cold buffer (25 mM HEPES, pH7.0 in 1× PBS) and washed by centrifuging again at 100,000 × *g* for 90 min at 4 °C. The washed pellet (1/10th volume of original supernatant) was designated as exosome-like particles. The protein concentration of the nanoparticles was measured and analyzed by Western blot analysis with EV-specific marker antibodies (positive markers: TSG-101 and CD63; negative markers: GM130 and Cytochrome c). The size, number, and morphology of exosome-like particles were characterized by nanoparticle tracking (NanoSight, Wiltshire, UK) and Transmission Electron Microscopy (TEM).

### Nanoparticle tracking analysis (NTA)

As previously described, [29] exosome-like particles were analyzed by NTA to measure the size, size distribution, and concentration of exosomes using a NanoSight LM10 system (Nanosight, LM10, Malvern Instruments, Worcestershire, UK). Briefly, each exosome-like particles sample was diluted in PBS containing 25 mM HEPES (pH 7.0) buffer until 50–100 nanoparticles could be tracked in the field of view of the NanoSight LM10 system. The setting of measurement parameters was also identical for all measurements. The camera level was set at 14 during the view-capturing process. The detection threshold was set at 6, and the screen gain was set at 8 during the video processing process. Five videos of 60 s duration were recorded for each sample.

### Transmission electron microscopy (TEM)

TEM analysis of the exosome-like particles was carried out as previously described [23]. The exosome-like particles were fixed with 2.5% glutaraldehyde in 0.1 M sodium cacodylate buffer. Samples are incubated on grids and negatively stained with 2% uranyl acetate. Imaging was performed on a Philips/FEI T-12 transmission electron microscope and analyzed by NIH ImageJ software.

### rST6G and exosome-like particles treatment to the cells

The day before treatment, cells were seeded in 50% confluency and cultured in a full serum medium. Then, cells were washed twice with serum-free medium and treated with recombinant rST6G (1–2 µg/ml) ± CMP-Sia for some experiment or equivalent vehicle, as indicated in the figures/legends. Exosome-like particles (1–2 µg/treatment) with or without pre-incubated with rST6G (5 min at room temperature) were added to the cells, as indicated in the figures/legends. After the treatments, cell proliferation, 3D spheroid assay, Western blots, and qPCR analyses were performed.

### Cell proliferation assay

Cell proliferation assays were carried out as described [30]. Briefly, the day before treatment, cells were cultured in full-serum medium on the 96-well tissue culture plate. The next day, cells were washed twice in a serum-free medium, and treatments were performed, as indicated in the figures/legends. Cell growth was also measured by adding WST-8 reagent and incubating at 37 °C for 1 h; absorbance was measured at 450 nm with background subtraction.

### 3D invasion assay

Cancer cell spheroid assays were performed in a 3D cell culture setting with modifications [31–34]. Briefly, 20,000 cells were cultured in DMEM F12 medium (Gibco-Thermo Fisher Scientific, NY, USA) supplanted with 20 ng/ml each of human EGF (Pepro Tech, NJ, USA), human FGF, and 1× B27 (Gibco-Thermo Fisher Scientific) in ultra-low attachment 24-well plates (VWR) for 24 h in the presence of 1–2 µg/ml rST6G, CMP-Sia, or equivalent vehicle controls. In addition, for some experiments, cells were grown in the presence of 1–2 µg self- or source cell exosome-like particles with or without rST6G, as indicated in the figure/legends. Then, cells were mixed thoroughly with the BD Matrigel (Biosciences, CA, USA) (2:3 ratio) in the DMEM F12-Growth Factors medium and plated on ultra-low attachment plates. Finally, tumor cell spheroids were treated with the rST6G or exosome-like particles, or equivalent vehicle control, as indicated in the figure/legends for another three days. 3D spheroids in 5 randomly selected fields were imaged at ×10 magnification using a Nikon Eclipse Ti-U Inverted Phase Contrast Microscope (Nikon Instruments Inc., NY, USA). The aggressive tumor cell invasion phenotypes were analyzed using ImageJ software (National Institute of Health, MD, USA). 3D spheroids invasion was quantified as the average number of invadopodia/protrusions per spheroid (*n* = 5–10). In addition, for some experiments, the spheroids invasion was quantified as the average length (µm) of invadopodia/protrusions per spheroid (*n* = 5–10).

### Immunofluorescence

Cells are grown onto 13 mm sterile glass slides overnight with 50% confluences in a 5% FBS-containing medium. For some experiments, the cells were fixed with 4% paraformaldehyde for 10 min at 37 °C; before paraformaldehyde-fixing, cells were washed twice with serum-free medium and treated with exosome-like particles ± rST6G or equivalent vehicle control, as indicated in the figure/legends. First, slides were permeabilized with 0.1% Triton X-100 (Sigma) for 5 min at room temperature (RT). Next, slides were washed with 1× PBS and blocked with 1% BSA (Sigma) for one hour at RT. After brief washing with TBS-T (0.1% Tween 20), slides were incubated with Direct FITC-conjugated SNA-lectins [35] (Sambucus nigra agglutinin, Vector Laboratories, CA, USA) at 1:500 dilution in 1% BSA containing TBS-T for two hours with mild rocking at RT. Finally, slides were washed extensively with TBS-T and stained with DAPI (BioLegend, CA, USA). Images were acquired with fixed exposure time and parameters and processed with the ZOE Fluorescent Cell Imager (Bio-Rad). Images processed by Fiji/ImageJ software (NIH) with four fields of view from two biological replicates were assessed [36]. SNA-lectins staining was expressed as mean pixel brightness (FITC staining only)/the number of DAPI-stained nuclei per image = mean fluorescence intensity (MFI) per cell [37].

### Immunohistochemical detection of ST6GAL1

We obtained de-identified TNBC clinical specimens from BDDR at RPCCC with Institutional Review Board approval (RPCCC, BDR # 074516). ST6GAL1 expression was evaluated by immunohistochemistry in the TNBC tissue section of one individual, as previously described [18]. The tissue specimen was embedded in an OCT compound (Leica Microsystems, Wetzlar, Germany), and cryosections of the embedded tissues were prepared. Slides were blocked in 5% BSA for 1 h, incubated overnight with anti-ST6GAL1 antibody, then with anti-goat-HRP secondary (R&D Biosystems) for 1 h. Tissues were then immersed in Impact DAB stain (Vector Labs) for 120 s and rinsed in water for 3 min. Images were acquired under 200× and 630× (insert) magnifications with a Nikon Eclipse E600 Optical microscope. Spot RT3 camera and Spot Software were used to capture and analyze the mages. Dark brown (red arrow) and light brown (green arrow) of ST6GAL1 stainings are indicated as a heterogeneous expression pattern of ST6GAL1 protein.

### Bioinformatics analysis

Transcriptome data was acquired from The Cancer Genome Atlas (TCGA) breast cancer cohort through cBioportal [38]. ST6GAL1 mRNA expression levels were compared between breast cancer (TCGA, *n* = 1078) and normal healthy breast tissues (*n* = 52). ST6GAL1 mRNA expression data were examined in breast cancer subtypes based on IHC (ER + HER2-, *n* = 584; HER2+, *n* = 181; TNBC, *n* = 159) in TCGA cohort. ST6GAL1 mRNA levels were also compared in different stages (stage 1, *n* = 178; stage 2, *n* = 610, stage 3, *n* = 245; stage 4, *n* = 19) of breast cancer patients in TCGA cohort.

The data set derived from the Molecular Taxonomy of Breast Cancer International Consortium (METABRIC) (*n* = 1904) was utilized to validate ST6GAL1 mRNA distribution data in subtypes, aggressive stages, and histological grades [39]. In METABRIC cohort ST6GAL1 mRNA distribution data was analyzed in breast cancer subtypes (ER + HER2−, *n* = 1355; HER2+, *n* = 236; TNBC, *n* = 313), stages (staged 1, *n* = 475; stage 2, *n* = 800, stage 3, *n* = 115; stage 4, *n* = 9), and histological grades (grade 1, *n* = 165; grade 2, *n* = 740; grade 3, *n* = 927). Mutant-allele tumor heterogeneity (MATH) score, a measure of intra-tumor heterogeneity, median cutoff (high vs. low ST6GAL1 mRNA, *n* = 539/group) was calculated through R/Bioconductor package “maftools”; efficient analysis, visualization, and summarization of (MAF) files from large-scale cohort-based cancer studies (https://www-biorxiv-org.gate.lib.buffalo.edu/content/early/2016/05/11/052662) [40].

### Gene Set Enrichment Analysis (GSEA) of hallmark gene sets for metastasis

Gene Set Enrichment Analysis (GSEA) was performed using software provided by the Broad Institute (http://software.broadinstitute.org/gsea/index.jsp) [41]. A collection of annotated gene sets for use with GSEA software can be found in the Molecular Signatures Database (MSigDB) (http://software.broadinstitute.org/gsea/msigdb). The result of the GSEA is the normalized enrichment score (NES), which reflects the degree to which a gene set is overrepresented at the top or bottom of a ranked list of genes. Statistical analyses were performed using R software (http://www.r-project.org/). Statistical significance was defined by a false discovery rate (FDR) of less than 25%, as GSEA software recommended.

### Statistical analysis

Quantitative data are presented as mean ± SE of three independent replicates. We have used a one-way ANOVA test for datasets containing multiple group comparisons and Tukey’s post hoc test for the family-wise error rate comparison. Association between variables (e.g., gene expression and mutation load scores) was determined using the Mann–Whitney *U* test. Kaplan–Meier method with log-rank test was used to compare survival curves between groups. All statistical tests were performed using the GraphPad Prism 8 software. *P* < 0.05 was considered statistically significant (*P < 0.05, **P < 0.01, and ***P < 0.001).

## Results

### ST6GAL1 is heterogeneously expressed and elevated in aggressive human breast cancers

**Fig. 1:**
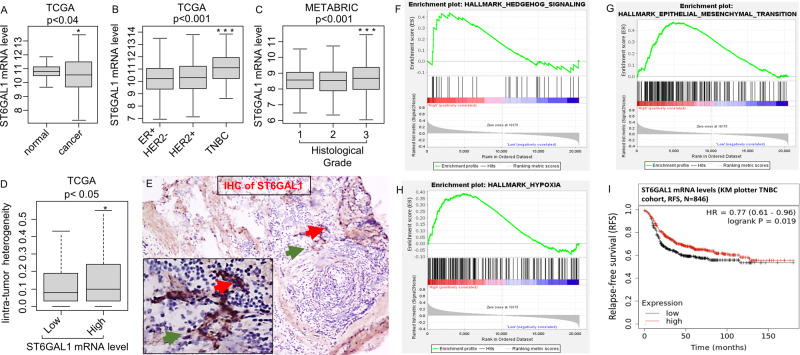
Elevated ST6GAL1 expression is associated with activating gene networks that promote a metastatic phenotype. A boxplot shows a high expression score of the ST6GAL1 gene in normal adjacent breast tissues (*n* = 117) vs. breast cancer patients of TCGA breast cancer cohort [64] with primary tumors (*n* = 979) (**A**). Student’s *t*-test, *p* = 0.031. Boxplots of the ST6GAL1 high expression score by immunohistochemistry (IHC) determined subtype in the TCGA breast cancer (**B**), and Nottingham pathological grades are shown for the METABRIC cohort [39] (**C**). All boxplots are Tukey type, and the boxes depict medians and inter-quartile ranges. One-way ANOVA and Tukey’s tests were used to calculate *p* values. Box plots of the intratumor heterogeneity [57, 58] in the TCGA breast cancer cohort by low and high ST6GAL1 score groups (**D**). Mann–Whitney *U* test and Kruskal–Wallis test were used to calculate the *p*-value. Median cut-off was used to divide two groups. IHC of ST6GAL1; representative (*n* = 3). **E** TNBC tissue section IHC with anti-ST6GAL1 antibodies are shown (red arrow higher and green arrow lower ST6GAL1 expression, respectively). Gene Set Enrichment Assay (GSEA) of high ST6GAL1 in the TCGA breast cancer cohort revealed enrichment in the Hedgehog (**F**), EMT (**G**), and Hypoxia (**H**) pathways. The normalized enrichment score (NES) and false discovery rate (FDR) values for TCGA-BRCA cohort are: Hedgehog, NES = 1.467799; FDR = 0.161496; EMT, NES = 1.458644; FDR = 0.160286; and Hypoxia, NES = 1.622797, FDR = 0.085881. FDR of 0.25 was used as the statistical significance of GSEA. A median cut-off was used to divide two groups (high vs. low ST6GAL1). **I** The Kaplan–Meier estimates the probability of relapse-free survival (RFS) by mRNA of TNBC patients. Analysis was provided by using the online KM-plotter [60].

TCGA, one of two gene expression databases with large breast cancer patient cohorts, was interrogated. Even though ST6GAL1 transcript abundance was diminished overall in breast cancer patients compared to matched controls (Fig. 1A), TNBC aggressive cancers have elevated ST6GAL1 mRNA compared to HER2+ and ER+ subtypes (Fig. 1B). In addition, METABARIC cohort analysis validated that ST6GAL1 expression was significantly elevated in TNBC and HER2+ compared with the ER+ subtype (Supplemental Fig. S1A).

Importantly, elevated ST6GAL1 mRNA was significantly associated with a higher tumor grade in breast cancer patients (Fig. 1C), even though ST6GAL1 expression does not appear correlated with the aggressive stage of breast cancer (Supplemental Fig. S1B, C). Importantly, elevated ST6GAL1 mRNA is associated with intra-tumor heterogeneity in breast cancer patients (Fig. 1D). When TNBC clinical specimens were examined by immunohistochemistry (IHC) using anti-ST6GAL1, we observed heterogeneous ST6GAL1 protein expression (Fig. 1E), similar to published results in other clinical cases [18, 42].

Overall, analysis using TCGA (Fig. 1F–H) and METABRIC cohorts (Supplemental Fig. S1D–F) revealed that elevated ST6GAL1 is associated with enrichment in gene networks associated with cancer stemness (Hedgehog), epithelial-mesenchymal transition (EMT), and hypoxia pathways. The data agree with the idea that ST6GAL1 is linked to the aggressive progression of breast cancers. Paradoxically in TNBC patients, an apparently opposing trend was observed, that higher ST6GAL1 expression is also associated with better relapse-free survival (RFS) (Fig. 1I).

### ST6GAL1 is heterogeneously expressed in breast tumor cells

**Fig. 2:**
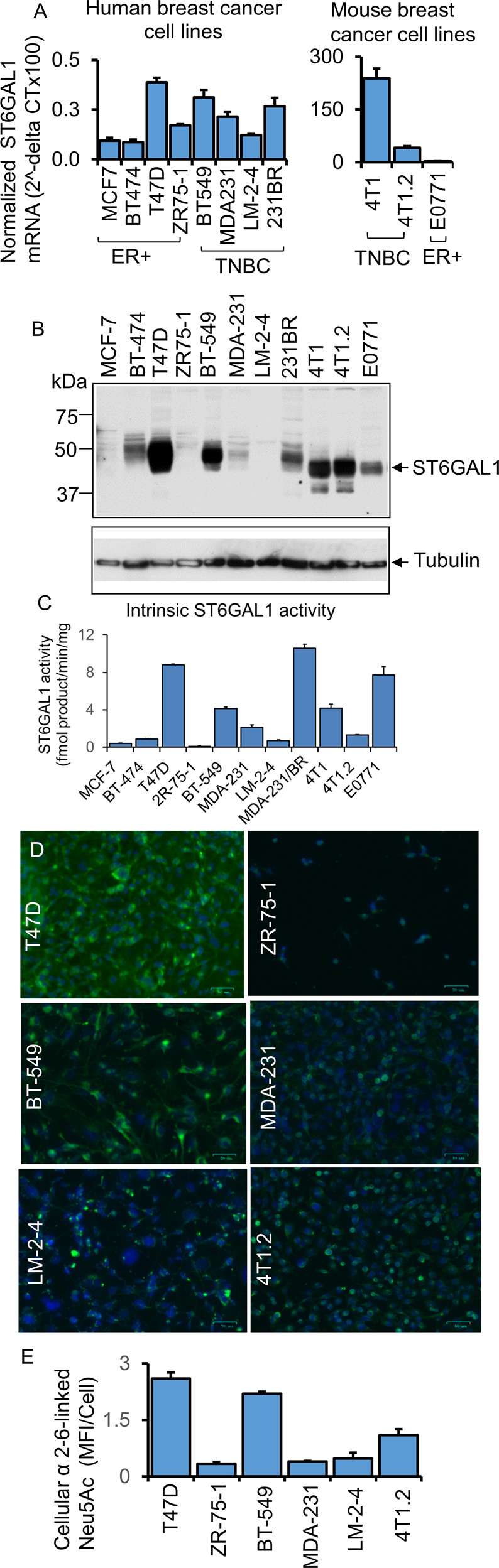
Functional ST6GAL-1 is variably expressed in breast cancer cells. **A** mRNA levels of ST6GAL1 were determined from ER + and TNBC human and mouse breast cancer cells, as indicated by quantitative real-time PCR (qPCR) and normalized to GAPDH (2^-delta Ct). *N* = 3, data are means ± s.e. **B** Total cell lysates from separate cell cultures (**A**) were used for Western blot analysis with antibodies against ST6GAL1. β-tubulin was used as a specific marker for cytosol and to show equal loading and transfer. **C** As indicated, an equal amount of proteins from cell lysates (**B**) was used for the ST6GAL1 enzyme assay, as previously mentioned [65]. ST6GAL1 activity is presented as fmol/min/mg protein; enzyme assays were performed in triplicates. Data are mean ± s.e. **D** Human and mouse representative ER+ and TNBC cell lines, as indicated, were immunostained with FITC- (green) labeled SNA lectin and DAPI (blue) for the nuclei before fixing. During fluorescent microscopy, exposure time and weighting for both DAPI and FITC fluorescence were kept consistent between samples. Representative images (*N* = 4) were shown on a scale bar of 50 µm. **E** Images were processed in ImageJ; background subtraction and MFI per cell calculations were carried out using the same parameters for each condition, and mean fluorescence intensity (MFI) per cell was shown with mean ± s.d. for four fields of view.

ST6GAL1 is a catalytically active enzyme that constructs the α2,6-sialic acid moiety on N-linked glycans of glycoproteins. To gain further insight into how ST6GAL1 expression is regulated that ultimately affects N-glycan sialylation in cancer, we examined an array of breast tumor cell lines (ER+ and TNBC) for ST6GAL1 expression on multiple levels of mRNA, protein, enzymatic activity, and by the abundance of the cognate enzymatic product, the α2,6-linked sialic acids on cell surface glycans (Fig. 2 panel A, B, C, D, E, respectively). Published data already suggested that various tumor cell lines, including breast cancer cell lines, have variable levels of ST6GAL1 protein [9, 11, 18].

Here, we examined an array of breast tumor cell lines (ER+ and TNBC). Two molecular forms of ST6GAL1 are generally observed, a larger 50 kDa and a smaller 42 kDa forms presumptively representing, respectively, the full-length and the smaller soluble catalytic domain is known to be proteolytically released from its N-terminal cytosolic and transmembrane anchor [18]. Among the panel of breast cancer cells examined, the human cells had the larger 50 kDa form exclusively (Fig. 2B). The observed electrophoretic mobility of the largest ST6GAL1 form in the three mouse cell lines examined (4T1, 4T1.2, and E0771) appeared somewhat smaller than the corresponding largest ST6GAL1 from the human cells. Presently, the molecular basis for this inter-species difference in electrophoretic mobility is not known, and presumably might be due in part to glycosylation differences on the ST6GAL1 protein. In two of the mouse breast cancer lines examined (4T1 and 4T1.2), a small amount of the lowest ~40 kDa form, presumably the catalytically released soluble catalytic domain, was also observed.

Figure 2 also illustrates that the levels of ST6GAL1 mRNA and protein were also highly variable within the cell line panel. Enzymatic assays were performed to measure the transfer of sialic acid from CMP-sialic acid to form Sia α2,6 joined to Gal(β1,4)GlcNAc-R (Fig. 2C). While there is general concordance between high ST6GAL1 protein with high ST6GAL1 catalytic activity amongst the cell lines examined, some notable differences were observed. Among the human cell panel, BT-474 had moderate ST6GAL1 protein presence but low detectable enzymatic activity. The mouse 4T1 and its metastatic variant 4T1.2 had roughly equivalent ST6GAL1 protein levels, but 4T1.2 had drastically less enzymatic activity than 4T1. The most striking discordance between protein and activity levels was in E0771, where high enzymatic activity levels but modest ST6GAL1 protein were observed. Moreover, MDA-231/BR also had the highest measured enzymatic activity but modest ST6GAL1 protein (compare Fig. 2B and C).

Cell surface α2,6-Sia structures were interrogated using SNA, the lectin from *Sambucus nigra* (Fig. 2D and E). In general, the observed cell-surface SNA reactivity aligned with the level of cellular ST6GAL1 mRNA and protein expression. Most striking, however, is the variable and heterogenous SNA reactivity amongst cells of the same strain. This is most pronounced in the lung-metastatic variant of MDA-231 cells, LM-2–4, but present in all cell lines examined, where only a subset of cells was SNA+ (Fig. 2D, E). ST6GAL1 mRNA also did not correlate entirely with the observed expression on the protein level (Fig. 2A). Notable are ZR75-1 and LM-2-4; both lines had intermediate mRNA levels but little to no detectable ST6GAL1 protein or enzymatic activity. These observations are consistent with the published data of human lymphoblastoma cell lines [18]. Furthermore, in agreement with the human breast cancer clinical cases, we observed that human breast tumor cells have very low levels (normalized 2^-delta CT) of ST6GAL1 transcripts at the steady-state culture conditions. Assuming that human and mouse primers for the ST6GAL1 are similarly efficient, mouse TNBC 4T1 and breast cancer bone metastatic 4T1.2 cells have higher ST6GAL1 transcripts than ER + E0771 cells (Fig. 2A, right panel).

Overall, the data describe highly variable expression of ST6GAL1 among breast cancer cell lines but also discordance between measurable enzymatic activity, ST6GAL1 protein, and mRNA levels among cells lines might be attributed to a poorly understood and largely unexplored complex differential kinetics of gene expression, protein translation, and longevity of the ST6GAL1 protein within the cells. As much of the intracellular ST6GAL1 is continually released into the systemic space, the levels of intracellular protein or measurable enzymatic activity might be highly dynamic and variable among different cells. Additional unknown factors, such as modifiers of intracellular ST6GAL1 mRNA and/or protein longevity, might also contribute.

### Transient knockdown of ST6GAL1 reduced breast tumor cell growth and invasiveness

During malignant progression, invasive cancer cells proliferate and penetrate the basement membrane by forming invadopodia structures that are enriched with actin filaments, adhesion proteins, and proteinases for tumor cell motility and degradation of the extracellular matrix (ECM) [43].

**Fig. 3:**
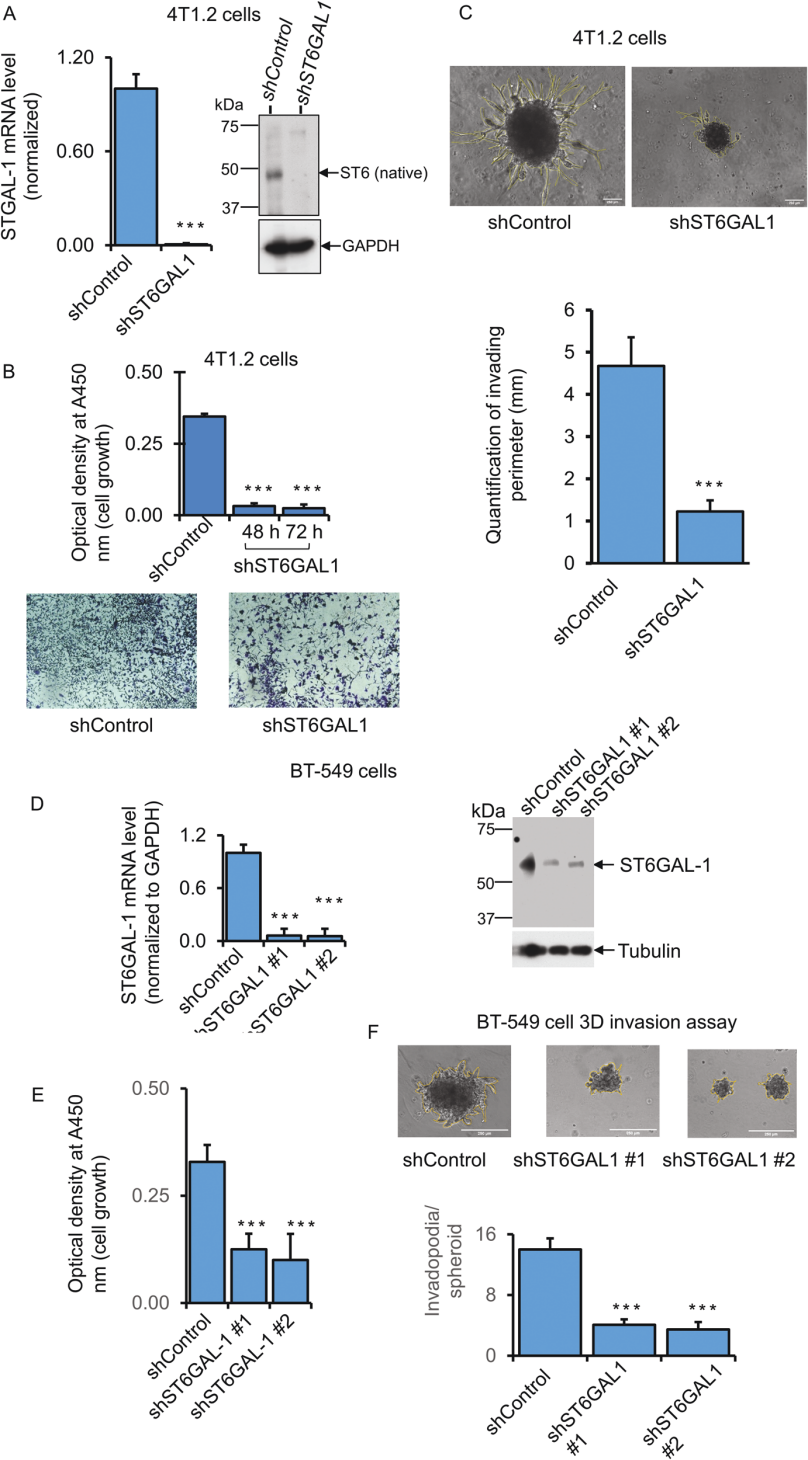
Natively expressing ST6GAL1 is involved in breast cancer cell growth and invasiveness. **B** Mouse breast cancer bone metastatic 4T1.2 cells were transfected with a validated shST6GAL1 #1 and shControl for 24 h with Lipofectamine P3000 reagents (Invitrogen), according to the company’s instruction. 5000 cells transfected with shControl and shST6GAL1 were cultured in the 24-well tissue culture plate in the medium containing 5% serum for another 48 h and 72 h, and cell proliferation was determined with WST-8 reagent. Normalized A450 nm readings were plotted (*n* = 5). Dara are means ± s.e., Student’s *t*-test, ***p < 0.001. A duplicate 48 h culture of 4T1.2 cells was used for Crystal Violet staining (**B**, lower panels). Representative live-cell images are shown for shControl and shST6GAL1. **A** Duplicate culture of 4T1.2 cells was used for SYBR-Green-qPCR and protein analyses for the ST6GAL1 gene. ST6GAL1 mRNA levels from the shControl and shST6GAL1 samples were normalized with the house-keeping gene GAPDH, and the normalized ST6GAL1 levels were calculated using the Delta-Delta Ct method, *N* = 3, data are means ± s.e., Student’s *t*-test, *p* < 0.001. A representative blot was shown for ST6GAL1 western blot analysis (*N* = 3), and GAPDH was used for housekeeping control for equal loading (**A**, right panels). (**C**, upper panels) 20,000 4T1.2 cells transfected with shST6GAL1 vs. shControl were plated on 12-well low-adherent tissue culture plates mixed with growth factor reduced Matrigel for 72 h to obtain aggressive tumor-cell Invasion in a 3D cell culture setting. The experiments were repeated three times, and representative phase-contrast images (×10 magnification) of tumor-cell invasions are shown for each condition; the yellow line indicates the invasion area on the spheroid body. (**C**, lower panel) Histograms represent the invading area’s quantification and the protrusion’s average length (*N* = 10 per condition, scale bar = 250 µm, data are ±s.e., Student’s *t*-test, *p* < 0.001. **D**–**F** Human TNBC BT-549 cells were transfected with shControl and shST6GAL1 #1 (Sigma Cat# TRCN0000035432) or shST6GAL1 #2 (Sigma Cat# TRCN0000035429), as mentioned before. 3D cell invasion assays (**F**, upper panels), quantification of cell invasion (**F**, lower panel), and cell proliferation (**E**) assays were performed. Duplicate cultures were used for qPCR and Western blot analysis of ST6GAL1 (**D**). For qPCR analysis, gene levels were normalized by GAPDH, *N* = 3, data are ±s.e., Student’s *t*-test, *p* < 0.05. Experiments were repeated at least three times, and representative blots and images were shown. For Western blotting, tubulin as a loading control for equal transfer. Histograms data for invasion assays are means ± s.e., scale bar 250 µm, one-way ANOVA, and post-hoc test for pair-wise comparison, *p* < 0.001.

To assess how ST6GAL1 expression might affect invasive cancer growth, a 3D spheroid culture method that closely mimics the in vivo environment, including a hypoxic tumor core, was used [33]. shRNA targeting was used to achieve significant knockdowns in several mouse and human breast cancer cell lines. The results for 4T1.2, E0771, and BT-549 are summarized. Cell growth and invasive characteristics were monitored in the shRNA knockdown cells (shST6GAL1) compared to cells transfected with random non-ST6GAL1 mRNA (shControl**)**. Figure 3 panel A shows that shRNA knockdown of 4T1.2 cells resulted in an almost complete reduction of ST6GAL1 mRNA and protein levels accompanied by dramatically reduced cell growth and almost complete attenuation invadopodia formation (Fig. 3 panel B, C, respectively). Supplemental Fig. S2 panel A-E shows the results for E0771 cells. Three distinct sets of guide shRNAs (shST6GAL#1, shST6GAL#2, and shST6GAL#3) targeting different sites of the ST6GAL1 mRNA sequence were used. All were effective in decreasing ST6GAL1 mRNA expression (Supplemental Fig. S2 panel A, C). shST6GAL#1 was evaluated to be highly effective in attenuation of cell proliferation; shST6GAL#2 and #3 had attenuated cell growth, although they were not as effective as #1 (Supplemental Fig. S2 panel B, E, respectively). ST6GAL1 protein levels were significantly decreased in #2 and #3 (Supplemental Fig. S2 panel D). The invasion was not measured in E0771 cells since they do not form invasive 3D spheroids in our cell culture. Figure 3D–F panels show BT-549 cells transfected with shST6GAL#1 and shST6GAL#2 guides or random control sequence (shControl). Both #1 and #2 guide RNAs effectively diminished ST6GAL1 mRNA and protein levels, compared to shControl, and concomitantly reduced cell growth and invadopodia formation (Fig. 3D, E, and F, respectively). For the most part, the decrease of ST6GAL1 mRNA in the knockdown cells generally parallels decreased intracellular ST6GAL1 protein levels. However, as mentioned earlier, there is a complex differential kinetics of gene expression, protein translation, and longevity of the ST6GAL1 protein within the cells that are poorly understood and poorly studied. Therefore, intracellular ST6GAL1 may be a transient pool that does not truly reflect the effectiveness of the knockdown.

### Breast cancer cells release ST6GAL1 in exosome-like vesicles

Cancer cell releases heterogenic extracellular vesicular small particles such as exosome (50–120 nm) and exomere (<50 nm) [23]. An enrichment of certain sialoglycoprotein and N-glycans [44], lectins [45], and neuraminidase [46] have been identified in exosomes, and adhesivity of exosome to target cells may be mediated at least in part by exosome glycans and carbohydrate-binding receptors [47]. In addition, colon cancer exosomes encapsulating ST6GAL1 that can be delivered to target cells and involved in cell signaling have been reported recently [23]. Here, we examine whether breast cancer cells also encapsulate functional ST6GAL1 into extracellular vesicles (EVs).

**Fig. 4:**
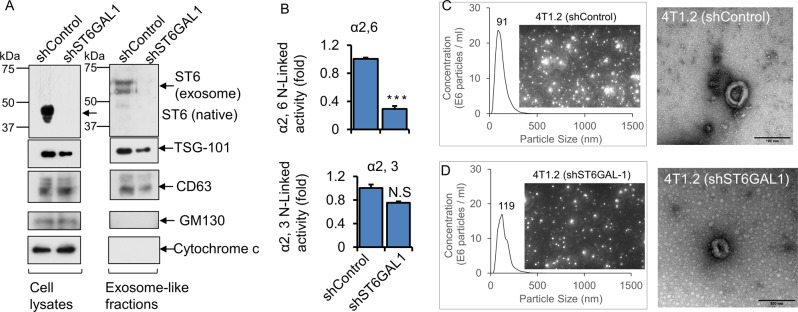
Breast tumor cells released exosome-like vesicles with heterogeneously expressed ST6GAL1. 4T1.2 cells were transfected with shControl or ShST6GAL1 #1 for 24 h, as mentioned above. Cells were cultured in the serum-free conditioned medium for another 48 h. **A** An equal amount of proteins from cell extracts (left panels) and exosome-like particles (right panels) were used for Western blot analysis with the indicated antibodies. Representative blots (*N* = 3) were shown. ST6GAL1 from cell lysates versus exosomes was analyzed in the same gel/membrane to compare their molecular size and presented in separate figures. Equal amounts of proteins from exosome fractions were used for α2,6 N-Linked activity (ST6GAL1) (**B**, upper panel) and α2,3 N-Linked activity (ST3GAL6)(B, lower panel) assays. Specific activity was calculated as fmol/min/µg specific product, plotted as fold activity, *n* = 3, data are means ± s.e., Student’s *t*-test, *p* < 0.001. NS; not significant. **C**, **D** Size distributions by nanoparticle tracking analysis (NTA) and images of exosome-like particles, which are screenshots from recorded videos of EVs when characterized by NTA. Exosome-like particles were isolated from shControl (**C**) and shST6GAL1 (**D**) transfected 4T1.2 cell culture-conditioned medium (100× dilution) and were examined by NTA. (**C**, **D**; right panel, respectively) Negative stain transmission electron microscopy (TEM) imaging of exosome-like particles. Representative images are shown. Scale bars: 100 nm.

Exosome-like vesicles were isolated from confluent monolayers of 4T1.2 breast cancer cells grown in a serum depleted medium for 48 h. The supernatants were centrifuged at 500 × *g*, 10,000 × *g*, and 100,000 × *g*, as previously described [23, 44]. 100,000 × *g* pellets were strongly enriched for EVs markers, CD63, Tsg101 but did not express the cis-Golgi matrix protein GM130 and mitochondrial localized protein Cytochrome c (Fig. 4A), as expected for an EV preparation without substantial contamination by cytosolic or mitochondrial protein [48]. 4T1.2 EVs cargo carries active ST6GAL1 as well as at least one other sialyltransferase ST3GAL6. shST6GAL#1 knockdown resulted in a ~4-fold decrease of ST6GAL1 in exosomes, but a negligible change in α2,3-sialyltransferase activity, most likely ST3GAL6, measured as normalized to EV protein concentration (Fig. 4B). Representative size distributions measured by nanoparticle tracking analysis (NTA) and transmission electron microscopy (TEM) images of EV from shControl and shST6GAL#1 are also shown (Fig. 4C, D). Supplementary Figure S3A–F shows size distributions of exosomes released by ER+ (T47D, ZR-75-1, E0771) and TNBC (BT-549, MDA-231, and 4T1.2) cancer cells. In general, the EVs are a heterogeneous population in the range between 50 and 400 nm particle sizes. The maximum of the major peak ranged between 86 and 128 nm of EVs in the six breast cancer cell lines tested. Concentrations of EVs are also varied based on the cell of origin. A cell line-dependent difference in nanoparticle secretion is similar to what has been previously reported [49].

ST6GAL1-knockdown in 4T1.2 cells resulted in strikingly reduced ST6GAL1 in exosomes (see Fig. 4A). The morphology of the exosomes measured by TEM was similar between shRNA-knock down and shControl samples (Fig. 4C, D). There was a slight reduction of exosomes concentration measured by NTA and exosomal marker proteins enrichment (e.g., Tsg101 and CD63) in shST6GAL1 compared with shControl cells. Overall, our observations point to breast cancer cells packaging ST6GAL1 into exosome-like structures that are released into the extracellular milieu. The level of ST6GAL1 packaged and released within exosome-like particles varies depending on the breast cancer cells in our panel.

### Cancer exosome-like particles potently amplify extrinsic ST6GAL1 to boost proliferation and invasiveness of breast cancer cells ex vivo

ST6GAL1 packaged within cancer exosomes can cell-surface sialylated and elicit signaling in recipient target cells [23]. Here, we demonstrate that extracellular ST6GAL1 elicits definitive phenotypic consequences in the recipient cells in the forms of enhanced cancer cell proliferation, growth, and invasive behavior.

**Fig. 5:**
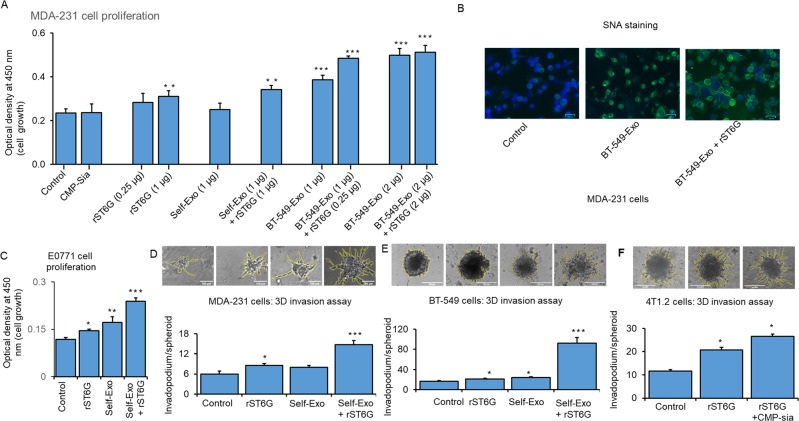
Extracellular ST6GAL1 enhances breast tumor cell proliferation and invasiveness. **A** Human breast cancer MDA-231 cells were treated with a control medium, 25 µM CMP-Sia, 0.25-1 µg/ml recombinant rat ST6GAL1 protein (rST6G), 1–2 µg exosome-like particles (BT-549) or 1 µg self-exosomes (MDA-231) as a control from a separate culture with or without additional rST6G, as indicated in serum-free medium for 48 h. Cell proliferation was measured by WST-8 reagent. *N* = 3, data are means ± s.e., ANOVA, post-hoc *t*-test, *p < 0.05, **p < 0.01, ***p < 0.001. **B** MDA-231 cells were treated with a control medium, 1 µg exosomes (BT-549), or exosome particles (BT-549) mixed with 1 µg rST6G for 20 min in a serum-free medium. SNA-lectins were stained (Green), and nuclei were stained with DAPI (blue color). Representative overlay images are shown on a scale of 50 µm. **C** Mouse breast cancer E0771 cells were cultured in the serum-free medium and treated with a control medium, 1 µg rST6G, 1 µg self-exosomes, or in combination, as indicated for 48 h. Cell proliferation was measured, as mentioned before. Mouse metastatic breast cancer 4T1.2 (**F**), human breast cancer MDA-231 (**D**), and BT-549 cells (**E**) were used for 3D spheroids assay with the indicated treatments, including rST6G only. Representative 10× magnification light microscopy images are shown. Histograms are the quantification of cancer cell invasion, *n* = 5; data are means ± s.e., ANOVA, post-hoc *t*-test, *p* < 0.05 vs. control.

Human TNBC breast cancer cells MDA-231, natively ST6GAL1-low and cell-surface SNA-dim, were used as targets. Adding recombinant ST6GAL1 (rST6G) but not self-exosomes to the culture medium enhanced MDA-231 cell growth; the addition of CMP-Sia, the ST6GAL1 donor substrate, alone did not affect any effect on MDA-231 proliferation. However, self-exosomes from a different culture of MDA-231 and additional rST6G have resulted in significant MDA-231 cell growth. In addition, adding exosomes that were recovered from the conditioned media of BT-549, natively ST6GAL1-high cells, resulted in significantly greater MDA-231 cell growth, which could be further enhanced in the presence of both BT-549 exosomes and rST6G. Maximal growth was observed when the added BT-549 exosomes were doubled. At the increased level of BT-549 exosomes, the additional presence of rST6G did not result in greater MDA-231 growth (Fig. 5A). These observations promote the idea that extracellular or extrinsic ST6GAL1 affects the biology of the TNBC cancer cell MDA-231. MDA-231 cell surface SNA reactivity was also increased in the presence of BT-549 exosomes; SNA signal was increased further when both BT-549 exosomes and rST6G were present (Fig. 5B), supporting the idea that extracellular ST6GAL1, in the forms of BT-549 exosome encapsulated ST6GAL1 as well as free recombinant enzyme, can act on target MDA-231 in the construction of α2,6-sialic acid epitopes.

Mouse E0771 cells with a medium level of native ST6GAL1 expression (Fig. 2) was examined next. E0771 cell proliferation was elevated with added rST6G to the culture medium, as well as self-exosomes (e.g., exosomes isolated from a separate culture of E0771 cells). Maximum E0771 proliferation was observed when both E0771 exosomes and rST6G were present in the culture medium (Fig. 5C). The synergistic effects of rST6G and exosomes (see Fig. 5B SNA reactivity and Fig. 5C) strongly suggest the presence of exosome cofactor (s) contributing to the extracellular ST6GAL1 mediated α2,6-sialylation of the target cell surface.

The mouse 4T1.2, the human MDA-231, and BT-549 cells that form invadopodia in an in vitro 3D culture setting were used to assess how extracellular ST6GAL1 might affect aggressive cancer cell’s invasiveness. In 4T1.2 cells, invadopodia formation was doubled when rST6G was included in the culture medium; invadopodia formation was enhanced further when CMP-Sia, the donor substrate to ST6GAL1, was also included (Fig. 5F). This observation supports the idea that extracellular ST6GAL1-mediated catalysis can drive invasive tumor behavior in vitro. Invadopodia formation of human BT-549 cells was enhanced marginally by the addition of self-exosomes (isolated from a separate culture of BT-549 cells), but invadopodia formation was dramatically enhanced in the presence of both exosomes and rST6G (Fig. 5E). Since BT-549 already has significant native ST6GAL1 expression, this observation further confirms the ability of extracellular ST6GAL1 in driving BT-549 biology and the putative existence of cofactor (s) in exosomes to augment extracellular ST6GAL1 action. MDA-231 cells with low endogenous ST6GAL1 further confirm that self-exosomes (isolated from a separate culture medium of MDA-231 cells) and additional rST6 but not self-exosomes alone significantly augment MDA-231 cell 3D invasiveness (Fig. 5D).

### Extracellular ST6GAL1 compensates cell-intrinsic native ST6GAL1 for invasiveness ex vivo

Earlier (Fig. 3), we showed that shRNA knockdown of native ST6GAL1 expression strikingly muted invadopodia formation in vitro. 4T1.2 cells subjected to shRNA knockdown of endogenous ST6GAL1 (shST6GAL1#1), compared to control knockdown using irrelevant RNA sequences (shControl), had strikingly diminished invadopodia formation.

**Fig. 6:**
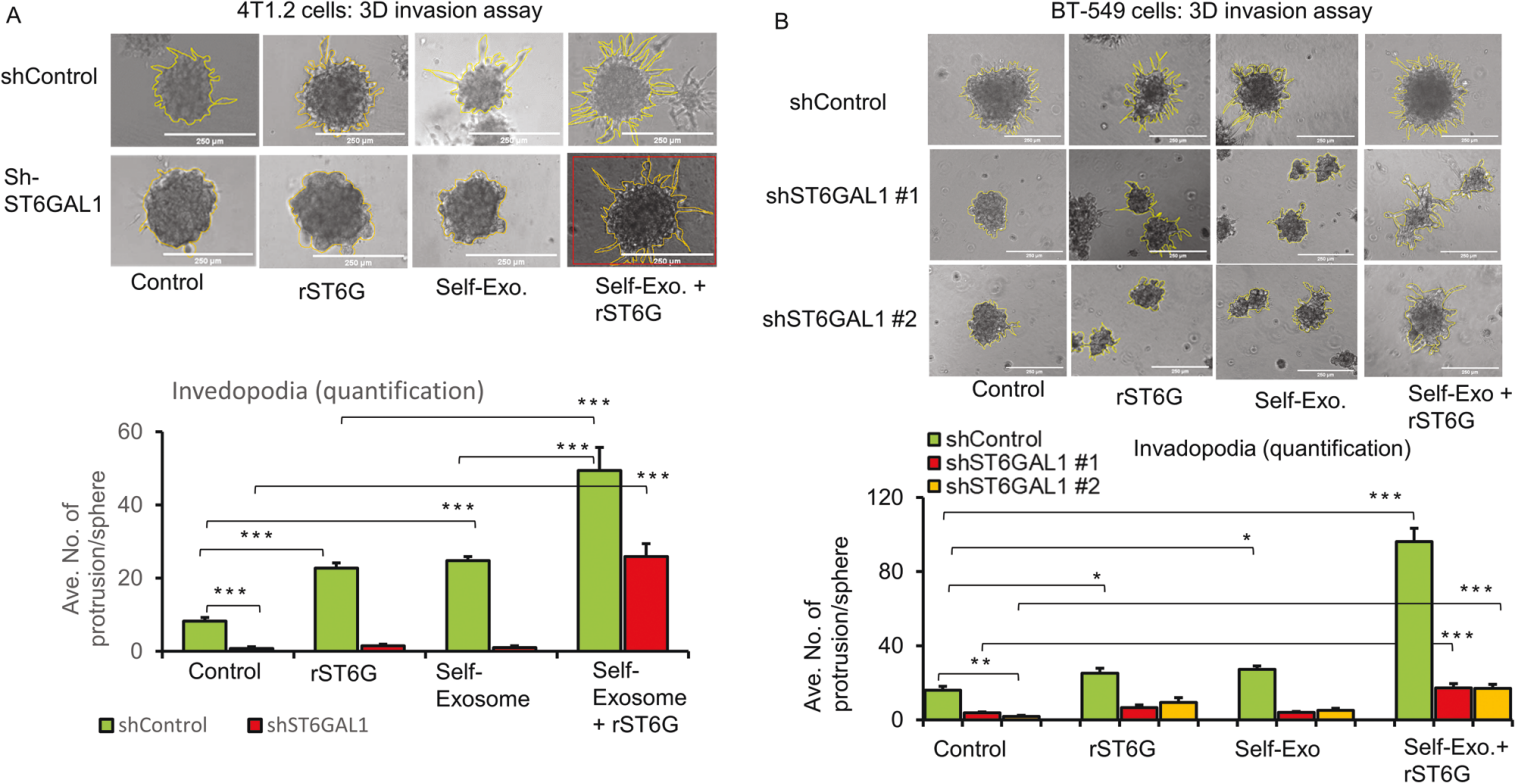
Extracellular ST6GAL1 compensates for cell-intrinsic ST6GAL1 actions in breast tumor cells. **A** 4T1.2 cells transfected with shControl or mouse shST6GAL1 #1 construct and invasion abilities were assessed with 3D spheroid cell culture setting after 48 h of treatments, as indicated. Representative images and quantification of cancer cell invasions are shown (**A**, upper and lower panel, respectively). Scale bar 250 µm, *N* = 5 spheroids, data are means ± s.e., ANOVA, post-hoc *t*-test, *p* < 0.001 vs. respective controls. **B** BT-549 cells transfected with shControl, human shST6GAL1 #1 or shST6GAL1 #2 were treated with the control medium, self-exosome particles, rST6G, or self-exosomes, rST6G in combination, and 3D invasion assays were performed. The scale bar 250 µm (**B**, upper panel) shows representative phase-contrast microscopy images. Quantification of invasions (**B**, lower panel), *n* = 5, data means ± s.e, ANOVA, post-hoc test, *p* < 0.0001 vs. respective controls. Separate cultures were used to validate knockdown efficiencies of ST6GAL1 in 4T1.2 and BT-549 cells by qPCR and Western blotting, Supplemental Fig. S5 panel A, B, respectively.

Here in Fig. 6A, we present evidence that invadopodia formation can be further enhanced in shControl, the cells with wild-type native ST6GAL1 expression when these cells are incubated with exosomes generated by a parallel shControl culture. This observation indicates a role for exosome contents in promoting invadopodia. In contrast, shST6GAL#1 exosomes are unable to restore invadopodia in the shST6GAL#1 spheroids, implicating the ST6GAL1 cargo within the exosomes as the driver for invadopodia formation. A prominent role for extracellular ST6GAL1 in invadopodia formation was confirmed when either shControl or shST6GAL#1 cultures were exposed to their respective self-exosomes with the further addition of rST6G. We observed the same dependence for extracellular ST6GAL1 for invadopodia formation in BT-549 cells (Fig. 6B). For BT-549, we show the results using two different sets of ST6GAL1 knockdown RNAs. As in 4T1.2, BT-549 exosomes from wild-type (shControl), but not from knockdown cultures, promoted invadopodia formation. The additional presence of recombinant ST6GAL1 further drives invadopodia formation. Supplementary Figure S5 panel A and panel B shows that shRNA knockdown of 4T1.2 cells and BT-549 resulted in an almost complete reduction of ST6GAL1 mRNA and protein levels. Additional breast cancer cells, 4T1 and E0771, were also tested, with similar results (Supplemental Fig. S4). Together these observations point to a role of extracellular ST6GAL1, either as encapsulated cargo within the exosomes or in freely soluble enzyme, in promoting invasiveness phenotype. Moreover, and most importantly, extracellular ST6GAL1 can be released from distal sources; non-cell-autonomous ST6GAL1 can compensate for the lack of native ST6GAL1 expression.

### Extracellular ST6GAL1 promotes enhanced expression of mesenchymal and stemness markers

**Fig. 7:**
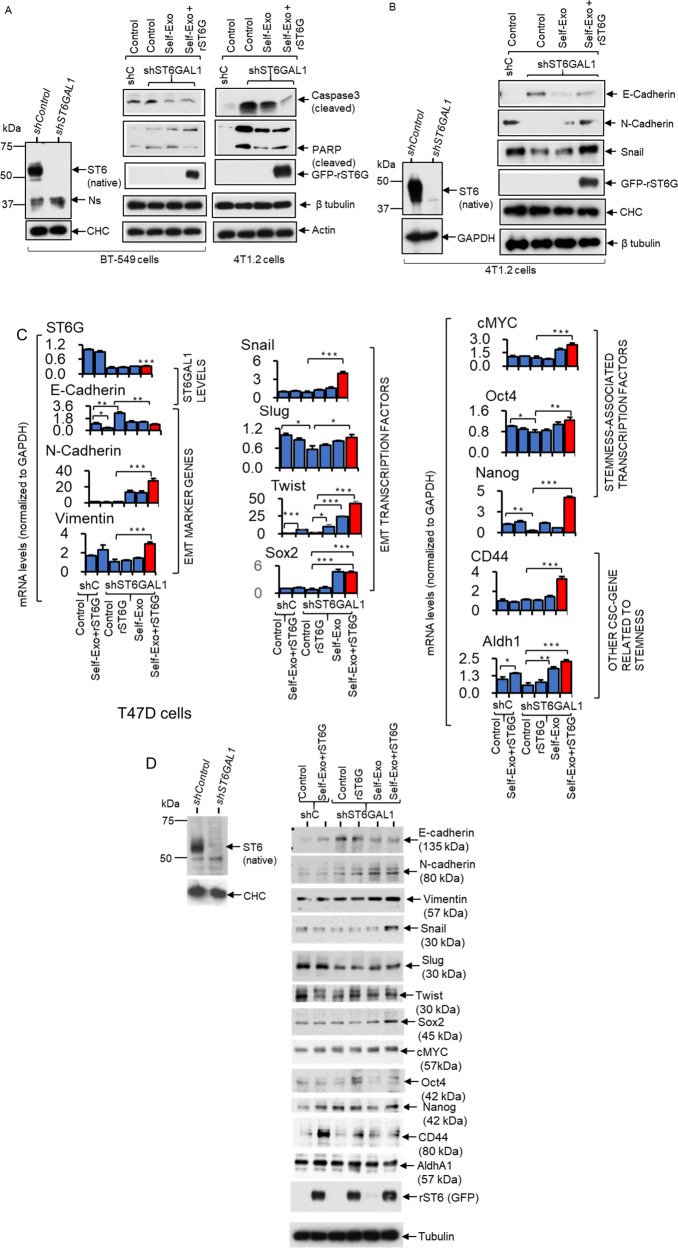
Extracellular ST6GAL1 regulates cancer stem cell transcription factors in breast tumor cells. **A** BT-549 and 4T1.2 cells, as indicated, transfected with the validated shST6GAL1 or the control shRNA were treated with the respective self-exosome particles or in combination with the rST6G for 48 h in a serum-free medium. **B** A separate culture of shControl and shST6GAL1 4T1.2 cells was used. Validated knockdown cells were treated with self-exosomes or in combination with rST6G, as indicated above. Cells extracts were used for Western blot analysis with the indicated antibodies. CHC, Tubulin, Actin, or GAPDH were used for equal loading transfer. Knockdown of ST6GAL1 in BT-549 and 4T1.2 cells was confirmed by Western blot of ST6GAL1, left panels of **A** and **B**, respectively. Ns in **A** (left panels) highlights a single non-specific band. Experiments were repeated at least three times; representative blots were shown. **C**, **D** Human breast cancer T47D cells transfected with the validated shST6GAL1 or the control shRNA were treated with the rST6G or combined with the self-exosome particles (shControl exosomes to shControl cells and shST6GAL1 exosomes to shST6GAL1 cells) for 48 h in serum-free medium. Cell cultures were used for SYBR-Green qPCR analysis with the indicated gene primers. Gene levels were normalized with GAPH and calculated by the delta-delta-Ct method. Data are means ± s.e., *n* = 3, ANOVA, post-hoc test, *p < 0.05, **p < 0.01 and ***p < 0.001. **D** Separate cultures of T47D cells were used for Western blot analysis with the indicated antibodies, including ST6GAL1 to validate the knockdown efficiency in shST6GAL1 versus shControl. Tubulin or CHC was used for equal loading and transfer. Experiments were repeated at least three times, and representative blots were shown.

Here, we examine the molecular contribution of ST6GAL1, particularly the ST6GAL1 in the extracellular spaces, on breast tumor cells. shST6GAL1 knockdown BT-549 and 4T1.2 cells were compared to their respective shControl, showing increased caspase 3 and PARP activation upon serum depletion (Fig. 7A), which is consistent with the idea that apoptosis is promoted in ST6GAL1 insufficiency. Moreover, self-exosomes (shST6GAL1 exosomes with diminished ST6GAL1 cargo) and additional rST6G compensated for diminished endogenous ST6GAL1 expression by dramatically suppressing cleaved Caspase 3 and PARP expression in both BT-549 and 4T1.2, supporting the idea that extracellular ST6GAL1 can compensate cell-endogenous STGAL1 and render protection from cell death (Fig. 7A). Extracellular ST6GAL1 also supplements cell-intrinsic ST6GAL1, driving mesenchymal and stemness marker expression. 4T1.2 cells with knockdown ST6GAL1 have elevated E-cadherin (an epithelial marker) and diminished N-cadherin (a mesenchymal marker), and decreased Snail (a mesenchymal associated transcription factor) and other mesenchymal stemness-associated markers (Fig. 7B and Supplemental Fig. S6). In T47D cells with a knockdown ST6GAL1, we observed increased E-cadherin (an epithelial marker) on the mRNA and protein level (Fig. 7C, D). The addition of self-exosomes together with rST6G dramatically suppressed E-cadherin expression, elevated N-cadherin, concomitant with elevated expression of EMT transcriptional factors Slug and Twist, and stemness-associated cMYC, Oct4, and Nanog. CD44 and Aldh1, other CSC genes related to stemness, were also elevated (Fig. 7C, D and Supplemental Fig. S6). Together, our observations support a role of ST6GAL1 in promoting the maintenance of the mesenchymal state, and ST6GAL1 insufficiency promotes a switch from mesenchymal to epithelial properties and decreases cell mortality. Most importantly, our data show extracellular ST6GAL1, not necessarily of cell-autonomous origins, can complement cell-intrinsic enzymes in maintaining mesenchymal phenotypes and survival.

## Discussion

The sialyltransferase ST6GAL1 modifies glycans and glycoproteins important not only in cancer progression [3, 8, 50] but also in the maintenance of cancer stem cell phenotype [51]. Generally, elevated ST6GAL1 and dysregulated α2,6-sialylation are prominently correlated with increased malignancy, especially in the lung, colorectal, pancreatic, and breast cancers [52, 53]. Canonically, ST6GAL1 resides in the intracellular ER-Golgi secretory complex and cell-autonomously sialylated nascent cell surface and secreted components. However, far less attention was paid to the extensive reservoir of catalytically active ST6GAL1 in the extracellular milieu, but published reports have implicated the extracellular ST6GAL1 as an important systemic regulator of blood cell development and function [16–19, 35].

A number of important conclusions can be drawn from our observations. First, the data suggest the importance of cancer cell ST6GAL1 in several important cancer cell phenotypes, including cell proliferation and invadopodia formation. Overall this is fully consistent with the known importance of ST6GAL1-mediated α2,6-sialic acid-linked glycans/glycoproteins in tumor progression and metastasis [1–3, 6, 9, 54].

Second, an extensive panel of mouse and human breast cancer cell lines had highly heterogenous ST6GAL1 expression. While we observed this heterogeneity is consistent with clinical breast cancer, how this heterogeneity contributes to overall malignancy and cancer progression is not yet clear. Breast cancer is a heterogeneous disease [55]. TNBC as the most aggressive and metastatic subtype of breast cancer and has limited therapeutic options. Aggressive metastatic diseases are killing more than 90% of breast cancer patients [56]. Therefore, understanding the role of sialylation in invasive, aggressive breast cancer is clinically relevant. Although ST6GAL1 transcripts levels are lower in breast tumors than in normal adjacent tissues in TCGA breast cancer cohort, however among cancer patients, ST6GAL1 transcripts levels are significantly elevated in triple-negative clinical cases compared with ER+ patients (Fig. 1A, B, and Supplemental Fig. S1A). By analyzing TCGA breast cancer data, we found that ST6GAL1 transcripts were not associated with aggressive stages of breast cancer in patients; however, they were linked to a higher grade of cancer patients (Fig. 1C and Supplemental Fig. S1B, C). In agreement with the cancer-promoting role of ST6GAL1, recent data revealed that ST6GAL1 is upregulated in metastatic pancreatic cancer cell models and displayed enrichment of gene networks associated with stemness, EMT, and hypoxia pathways [9]. Gene enrichment data analysis using both the METABRIC and TCGA breast cancer cohort suggested that higher levels of tumor ST6GAL1 transcripts were linked with metastatic progression of cancer pathways gene sets, including Hedgehog, EMT, and hypoxia (Fig. 1F–H and Supplemental Fig. S1D–F). In sync with the notion that ST6GAL1 is critical for breast cancer, we found that knockdown of ST6GAL1 with shRNA targeted to the different sites of ST6GAL1 significantly reduced aggressive breast cancer cell proliferation and invasive properties in a 3D cell culture setting (Fig. 3). We also observed that tumor ST6GAL1 transcripts are significantly associated with higher intra-tumor heterogeneity scores in breast cancer patients in TCGA breast cancer cohort (Fig. 1D). Poorly understood intra-tumor heterogeneity, with reference to the tumor-specific subclonal driver gene mutations and copy number alteration, is thought to be involved in aggressive cancers [57, 58]. ST6GAL1 might just provide another layer of heterogeneity in aggressive breast cancer.

Generally, there is a correlation between ST6GAL1 mRNA and protein in tumor cells; however, there is an exception [18]. Transcriptional regulation of the st6gal1 gene is complex in tumor cells, and we have noticed that human breast tumor cells have a low level of ST6GAL1 mRNA compared with mouse breast cancer cells. In some experiments, we have also noted that the level of intracellular ST6GAL1 protein does not always correspond with mRNA levels in breast cancer cells. Since most of the ST6GAL1 is continually secreted, it is likely that decreased mRNA will principally affect released ST6GAL1 rather than the retained enzyme. Indeed, that is what we observed in some shRNA-mediated ST6GAL1-knockdown experiments in breast cancer cells. However, we feel a study of the kinetic relationship between released and retained ST6GAL1 is beyond the scope of this manuscript and was not pursued here.

Third, breast cancer cells release bioactive exosome- and exomere-like particles, but the particles from different breast cancer lines have vastly different amounts of ST6GAL1 cargo. This is in agreement with earlier findings that released cargo of exosome particles are enriched with α2,6-sialylated glycoproteins [59] and ST6GAL1 [23]. Earlier studies in colon cancer cell models suggested that variable functional ST6GAL1 in exosomes released by tumor cells are linked to the source cells [23]. The study also suggested that tumor cells with a higher level of endogenous ST6GAL1 protein released exosomes can transfer ST6GAL1 into the lower natively expressing ST6GAL1 tumor cells and enhance ST6GAL1-mediated cellular signaling in the recipient cells. Our early work suggests that the liver mainly releases soluble ST6GAL1 that is found in circulation. Here, our data suggest that certain cancer cells have the capacity to significantly raise the local extracellular ST6GAL1 levels. In the form of a recombinant protein (rST6GAL1) in combination with the exosomes released by tumor cells, these are capable of impacting target tumor cells and compensates for endogenous ST6GAL1 functions. Fourth, the exosome ST6GAL1 cargo compensates for deficiency in cell-native ST6GAL1 expression in restoring proliferation and invadopodia formation. In the TNBC clinical specimens, we observed that ST6GAL1 is heterogeneously expressed (Fig. 1E), which could correlate with the heterogeneous expression of α2,6-sialic acid-linked lectins [11]. The biology driving this α2,6-sialylation heterogeneity is unknown, and how it contributes to the overall pathobiology of the cancer is also completely unknown. The heterogeneity may underscore the current confusion of why ST6GAL1 expression is observed to be beneficial but sometimes detrimental to patient outcome. Using KM plotter web-based data analysis [60], our results suggested that tumor ST6GAL1 mRNA was associated with favorable disease-free survival of TNBC patients (Fig. 1I), suggesting a complex mechanism of ST6GAL1-mediated cancer progression, warrant further investigation involving tumor microenvironment (TME). The ability of extracellular ST6GAL1 to compensate for the deficiency in cell-native ST6GAL1 expression may provide a new perspective in understanding the role of this sialyltransferase in cancer.

Fifth and most unexpectedly, although cancer exosomes devoid of ST6GAL1 cargo are unable to promote tumor cell survival, cell proliferation, and invadopodia formation, these properties are restored upon the addition of soluble recombinant ST6GAL1 (Fig. 6 and Supplementary Fig. S4). Notably, the addition of exogenous sources of ST6GAL1 can induce a more mesenchymal and CSC phenotype of breast cancer cells (Fig. 7 and Supplemental Fig. S6). This observation strongly implicates a cofactor in exosomes facilitating extracellular ST6GAL1 action. While the identity of this cofactor and its mechanism of action remains to be formally elucidated, an earlier report indicated that activated platelets release vesicle-like particles that supply the sialic acid donor substrate needed for the extracellular sialyltransferase catalysis [35].

Taken together, our data further support a role for ST6GAL1 in breast cancer progression. Breast cancer is a heterogeneous disease with multiple subtypes. We were using multiple TNBC and ER+ cell lines to establish that extracellular ST6GAL-1 mediated novel signaling is important for tumor cell survival, proliferation, invasion, and EMT progression. Canonically, ST6GAL1 resides within the intracellular ER-Golgi secretory complex, where the sialyltransferase cell-autonomously glycosylates nascent glycoproteins in biosynthetic transit. However, our data demonstrate the existence of an extracellular mechanistic pathway for ST6GAL1 function in breast cancer. At this time, it is unclear whether the intrinsic and extrinsic mechanisms are redundant pathways or whether these mechanisms address exclusive needs unmet by the other. Moreover, mechanistically it is not clear whether the external ST6GAL1 needs to be internalized by the target cells or the external ST6GAL1 acts directly on the cell surface of the targets. A recent study has elegantly demonstrated that tumor cells secrete ST6GAL1 in exosome vesicles or exomere smaller particles and are capable of transferring into the recipient cell as a functional ST6GAL1 in colon cancer cell models [23, 61].

On the other hand, other reports highly suggest that extracellular ST6GAL1 directly sialylates the cell surfaces of target cells [16–18, 35, 62, 63]. Whichever the mechanistic conduit for extracellular ST6GAL1 action, at least some biologic functions, namely promoting cell proliferation and invasiveness, can be restored by extracellular ST6GAL1 in lieu of adequate cell-native ST6GAL1 expression. While our data suggest the role of ST6GAL1 in driving aggressive cancer cell behavior, our data also indicate that the intrinsic ability of individual cancer cells to cell-autonomously express ST6GAL1 might not be absolutely necessary. Lastly, there is also the intriguing possibility that cancer cell natively expressed ST6GAL1 needs to be first excreted into the extracellular space.

## Supplementary information

Supplemental Figures with Legends (Fig 1-6)-Revised

Original data sets (WBs and qPCR data)

## Data Availability

Publicly available datasets were analyzed in this study.
