# Supplementary material for: Extracellular sialyltransferase st6gal1 in breast tumor cell growth and invasiveness
Source: Cancer Gene Ther. Author manuscript; Available in PMC 2022 Nov 17. (PMC9663294; doi:10.1038/s41417-022-00485-y)
Supplement: ExtracellularFigures [file NIHMS1841107-supplement-ExtracellularFigures.pdf]

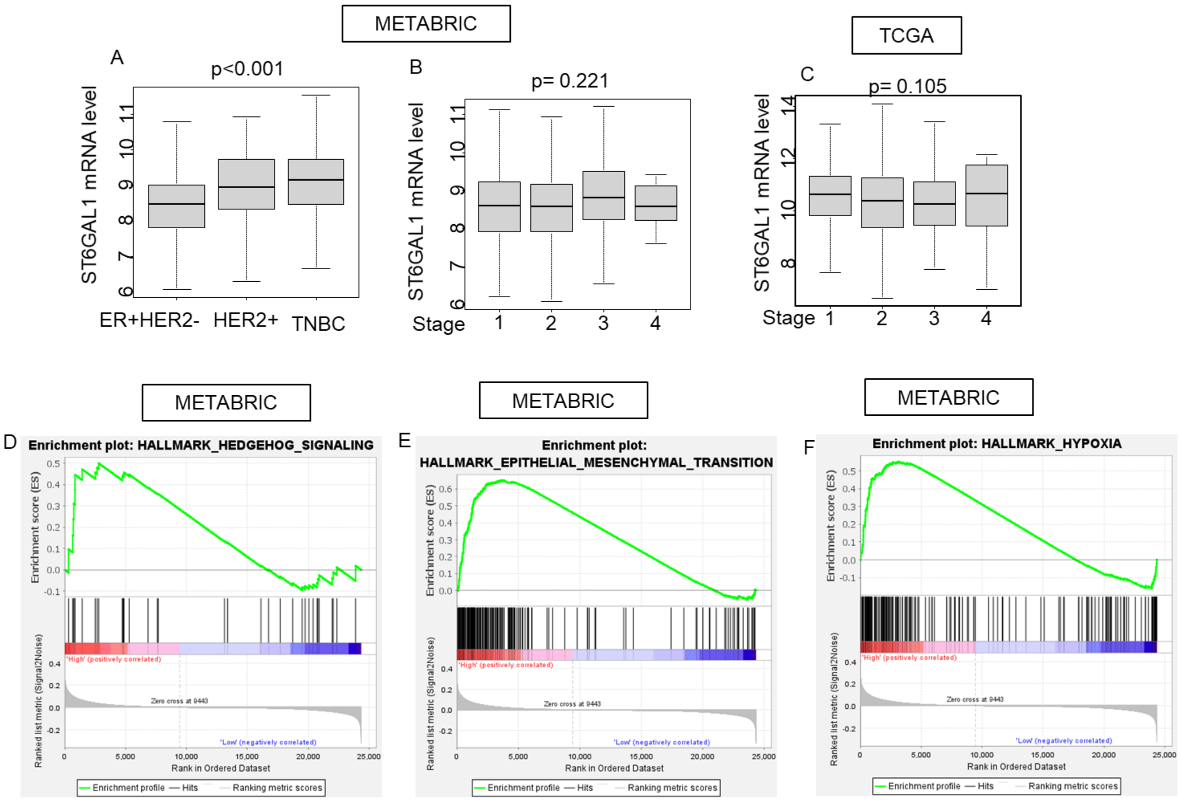

Supplemental Figure S1. ST6GAL1 is upregulated in aggressive subtypes among breast cancers and displays enrichment in gene networks associated with stemness and EMT. Boxplots of the ST6GAL1 high expression score by immunohistochemistry (IHC) determined subtype in the METABRIC cohort (A). Boxplots of high expression of ST6GAL1 score of tumors of different American Joint Committee on Cancer (AJCC) stages for METABRIC (B) and TCGA breast cancer (C) cohorts. All boxplots are of Tukey type, and boxes depict medians and inter-quartile ranges. One-way ANOVA and Tukey's tests were used to calculate p values. GSEA of high ST6GAL1 in the METABRIC cohort also revealed enrichment in the Hedgehog (D), EMT (E), and Hypoxia (F) pathways. The NES and FDR values are: Hedgehog, NES = 1.233785; FDR = 0.221941; EMT, NES = 1.314207; FDR = 0.192539; and Hypoxia, NES = 1.438181, FDR = 0.097375. FDR of 0.25 was used as the statistical significance of GSEA.

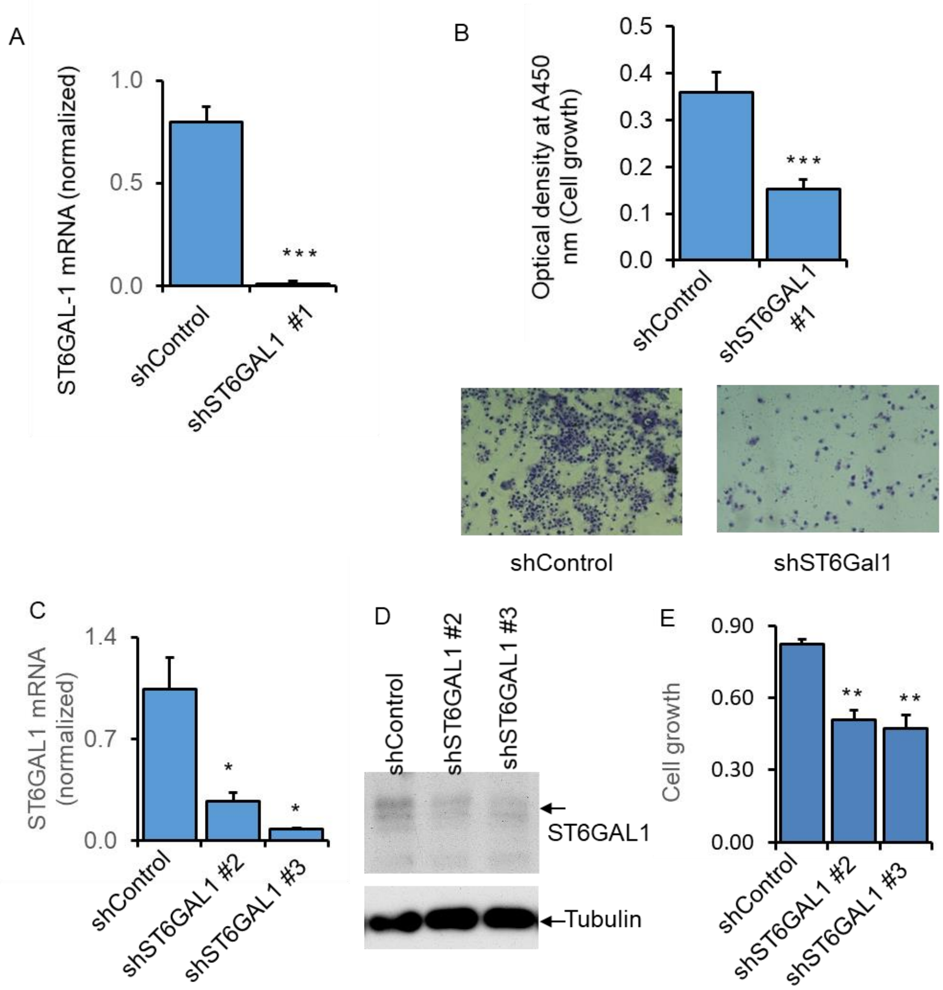

Supplemental Figure S2. Natively expressing ST6GAL1 is involved in breast cancer cell growth (A-E). Mouse breast cancer E0771 cells were transfected with shControl and shST6GAL1 #1 (A, B), or (C-E) shST6GAL1 #2 (Sigma Cat# TRCN0000018819) and shST6GAL1 #3 (Sigma Cat# TRCN0000018821) and cell proliferation (WST-8 assay)(B, E) and live cells staining (Crystal violet assay) were determined (B, lower panels). (A, C) Duplicate cultures were used for qPCR analysis for the ST6GAL1 gene. ST6GAL1 mRNA levels were normalized with GAPDH and plotted. N=3, data are ±s.e, Student's t-test, $p<0.05$ (*), $p<0.01$ (**), and $p<0.001$ (***). (D) Cell extracts from shControl or shST6GAL1 samples were used for Western blot analysis with anti-ST6GAL1 antibodies, and β-tubulin was used for equal loading and transfer. Representative blots were shown, n=3.

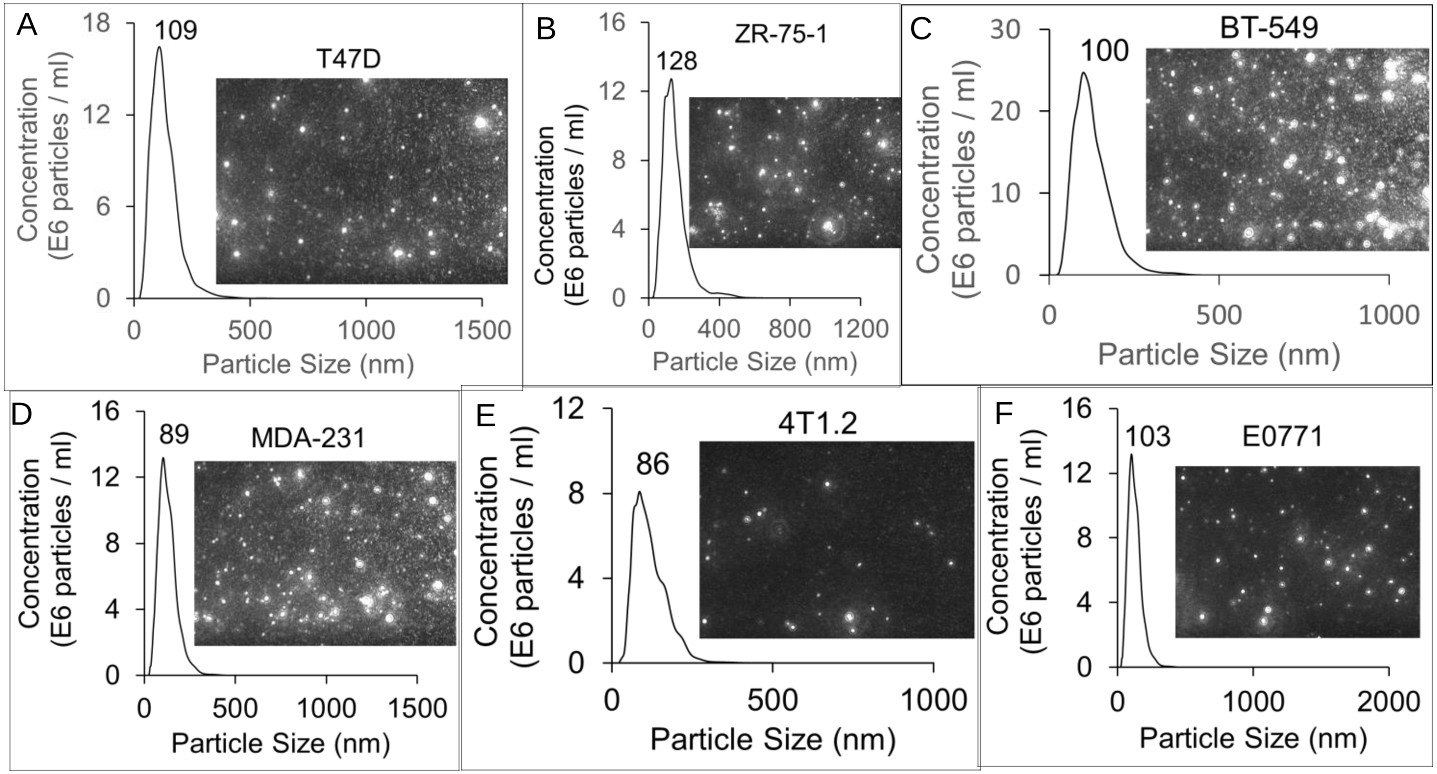

Supplemental Figure S3. Breast cancer cells released heterogeneous exosome-like particles. (A-F) NTA analysis and images of exosome-like particles isolated from breast cancer cells grown in the conditioned medium, as labeled.

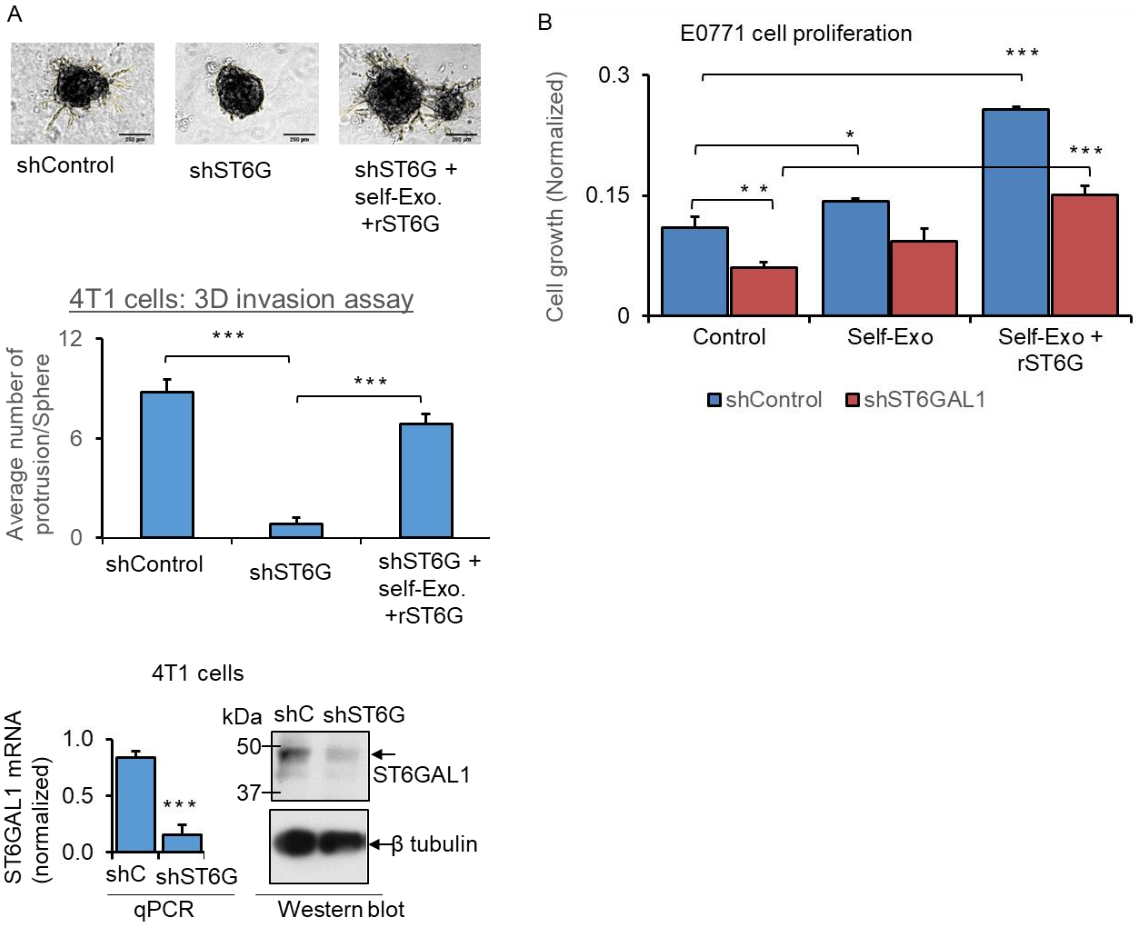

Supplemental Figure S4. Extracellular ST6GAL1 compensates for intracellular ST6GAL1 for breast cancer cell growth and invasiveness. (A) 4T1 cells transfected with shRNAs, as mentioned in Figure 6A, B, invasion abilities were assessed after 7 d of treatments. Representative images and quantification of cancer cell invasions are shown (A, upper and middle panel, respectively). Scale bar 250 µm, N=7 spheroids, data are means ± s.e, ANOVA, post-hoc t-test, p<0.001 vs. respective controls. As mentioned before, qPCR and Western blot analysis of ST6GAL1 in 4T1 cells. (A, lower panels; left and right, respectively). (B) E0771 cell proliferation assays with the indicated treatments for 48 h. Data are means of ± s.e, n=5, ANOVA, post-hoc test, p<0.01 (**) and p<0.001 (***) vs. respective control, as indicated.

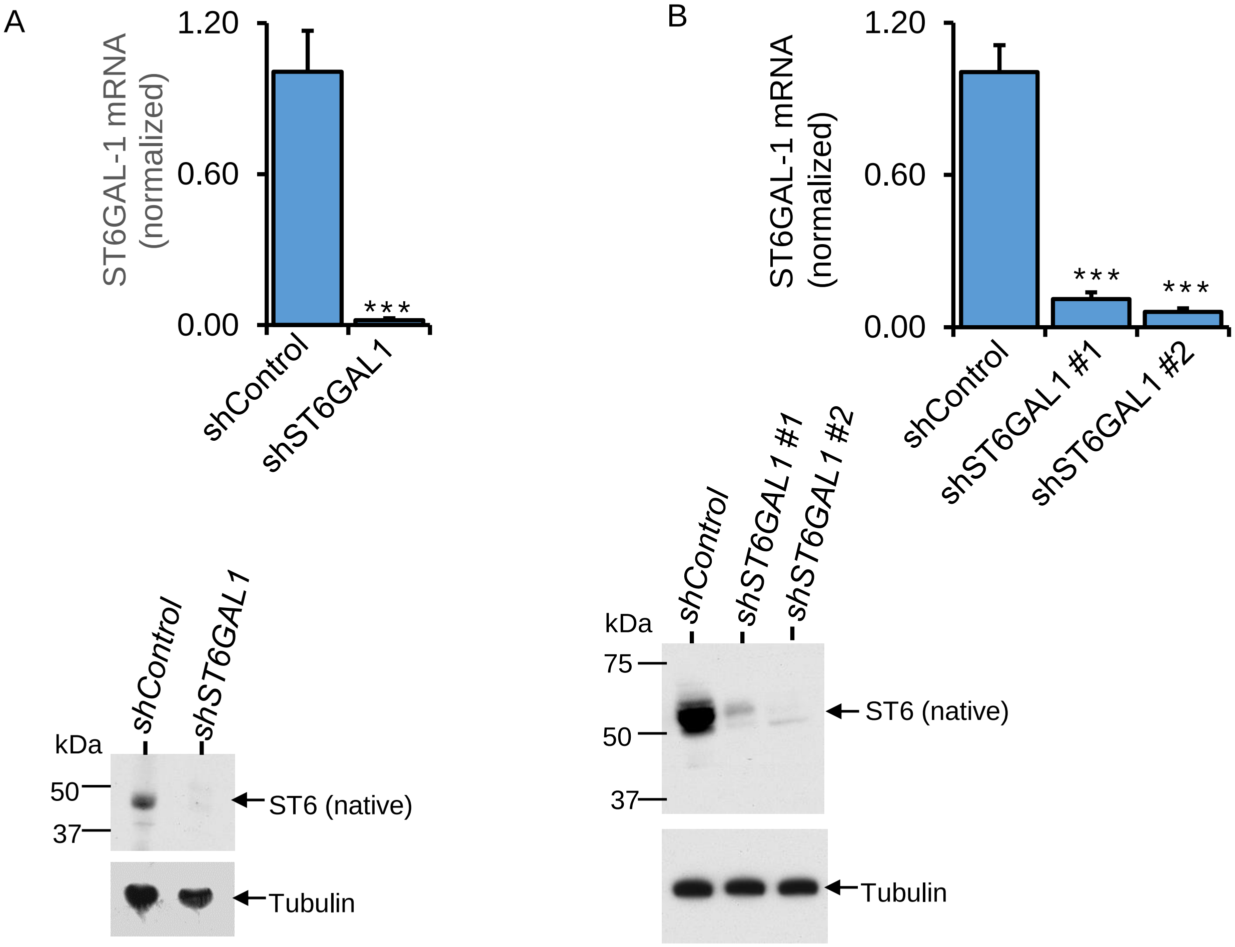

Supplemental Figure S5. Knockdown of ST6GAL1 in breast cancer cells. (A, B) Duplicate cultures of Figure 6A (4T1.2 cells) and Figure 6B (BT-549 cells) were used for SYBR-Green-qPCR and protein analyses for the ST6GAL1 gene. ST6GAL1 mRNA levels from the shControl and shST6GAL1 samples were normalized with the house-keeping gene GAPDH, and the normalized ST6GAL1 levels were calculated using the Delta-Delta Ct method, N=3, data are means ±s.e, Student's t-test, $p<0.001$ (A, B; upper panels). A representative blot was shown for ST6GAL1 western blot analysis (N=3), and Tubulin was used for housekeeping control for equal loading (A, B; lower panels).

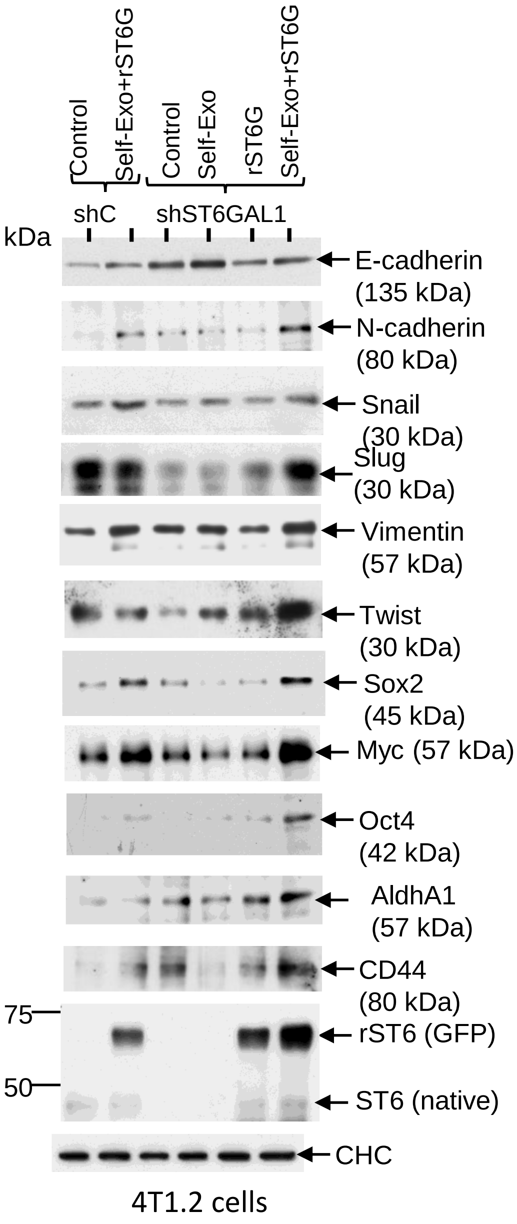

Supplemental Figure S6. Extracellular ST6GAL1 regulates EMT in breast cancer cells. (A) 4T1.2 cells were transfected with shRNAs, as mentioned in Figure 6A. shST6GAL1 or the control shRNA cells were treated with the rST6G or combined with the self-exosome particles (shControl exosomes to shControl cells and shST6GAL1 exosomes to shST6GAL1 cells) for 48 h in serum-free medium. Cell extracts were used for Western blot analysis with the indicated antibodies, including ST6GAL1. CHC was used for equal loading and transfer. Experiments were repeated at least 3 times, and representative blots were shown.
