# Supplementary material for: Extracellular sialyltransferase st6gal1 in breast tumor cell growth and invasiveness
Source: Cancer Gene Ther. Author manuscript; Available in PMC 2022 Nov 17. (PMC9663294; doi:10.1038/s41417-022-00485-y)
Supplement: ExtracellularDataSets [file NIHMS1841107-supplement-ExtracellularDataSets.pdf]

Figure 2B

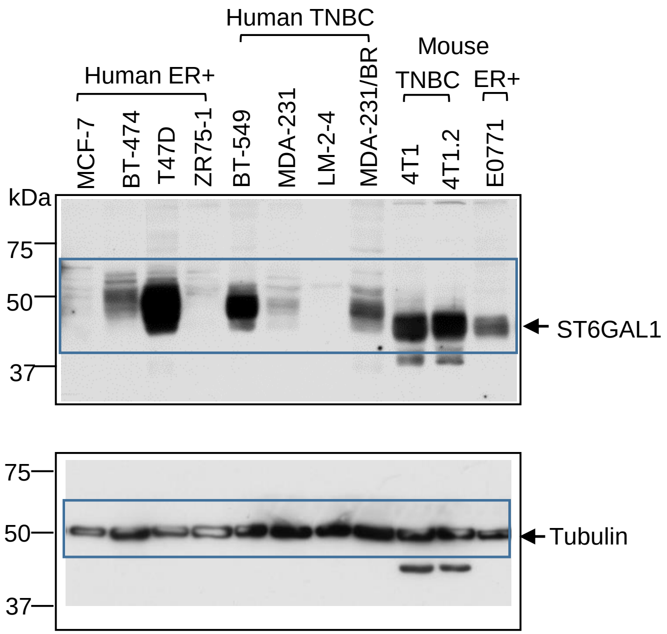

Figure 3A

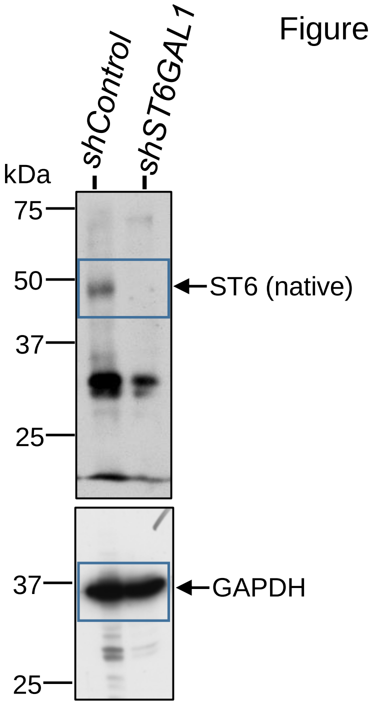

Figure 3D

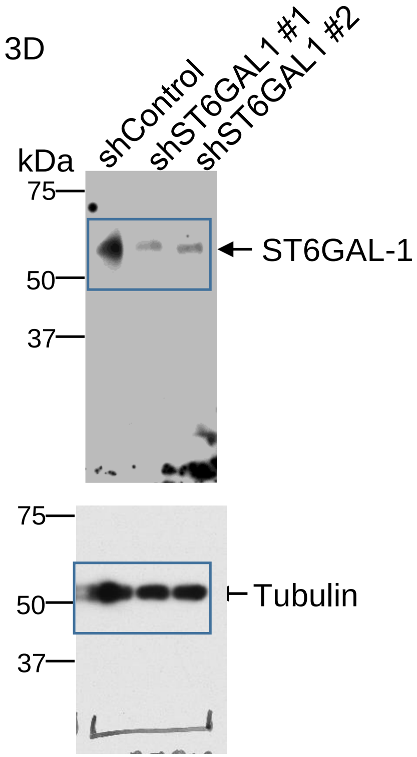

Fig 4A

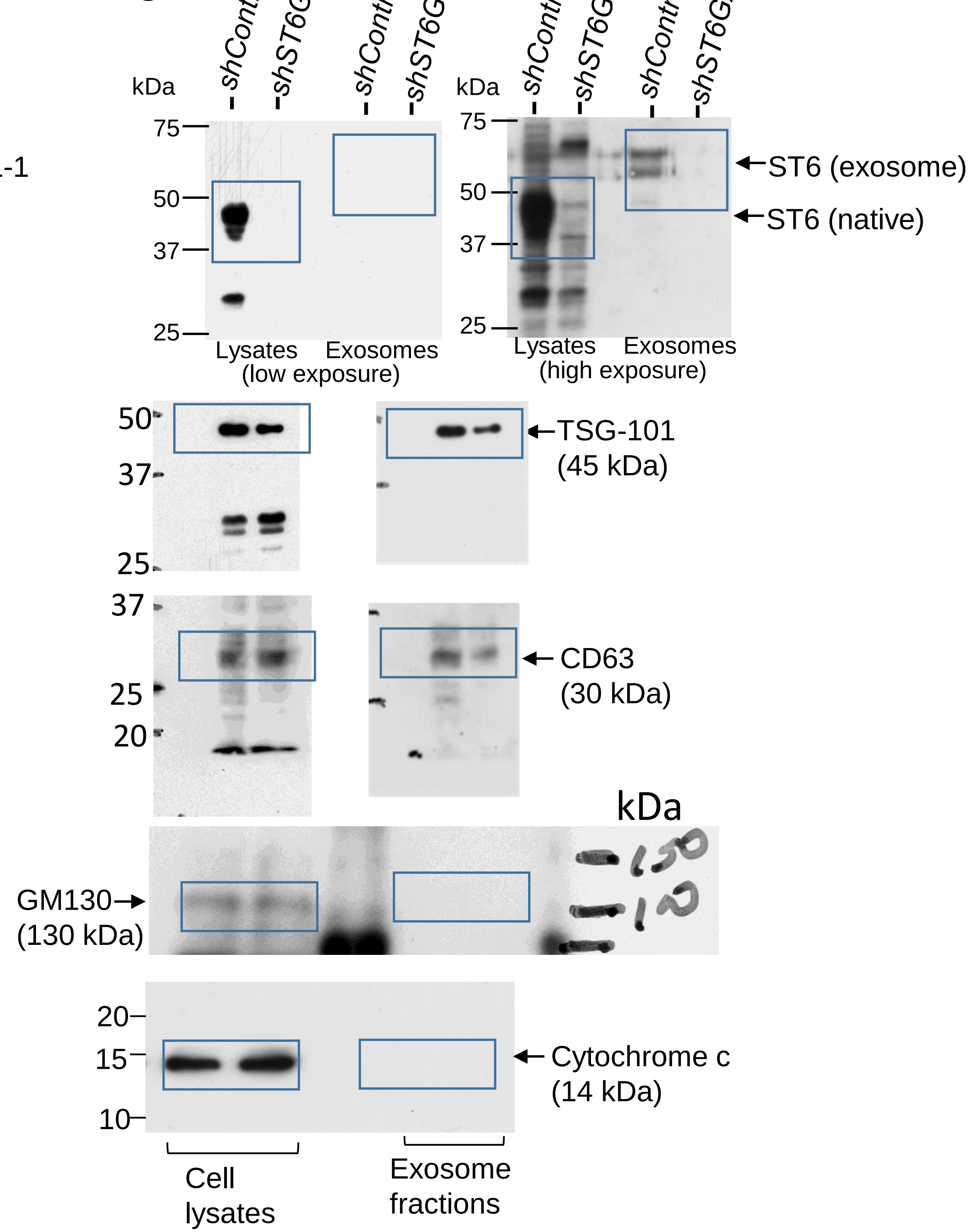

Fig. 7A

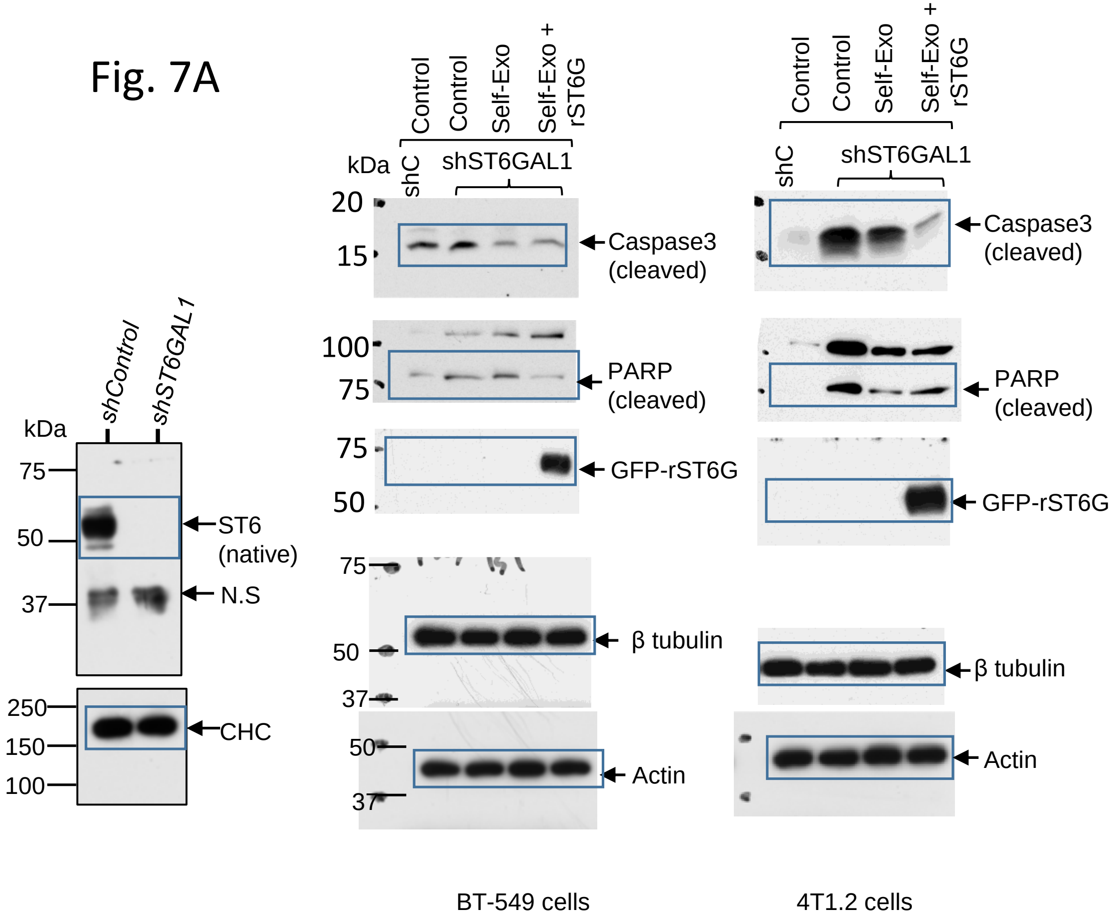

kDa
shControl
shST6GAL1
75
50
37
ST6 (native)
N.S
250
150
100
CHC
shC
Control
Control
Self-Exo
Self-Exo + rST6G
shST6GAL1
20
15
Caspase3 (cleaved)
100
75
PARP (cleaved)
75
50
GFP-rST6G
75
50
37
β tubulin
50
37
Actin
BT-549 cells
4T1.2 cells

Fig. 7B

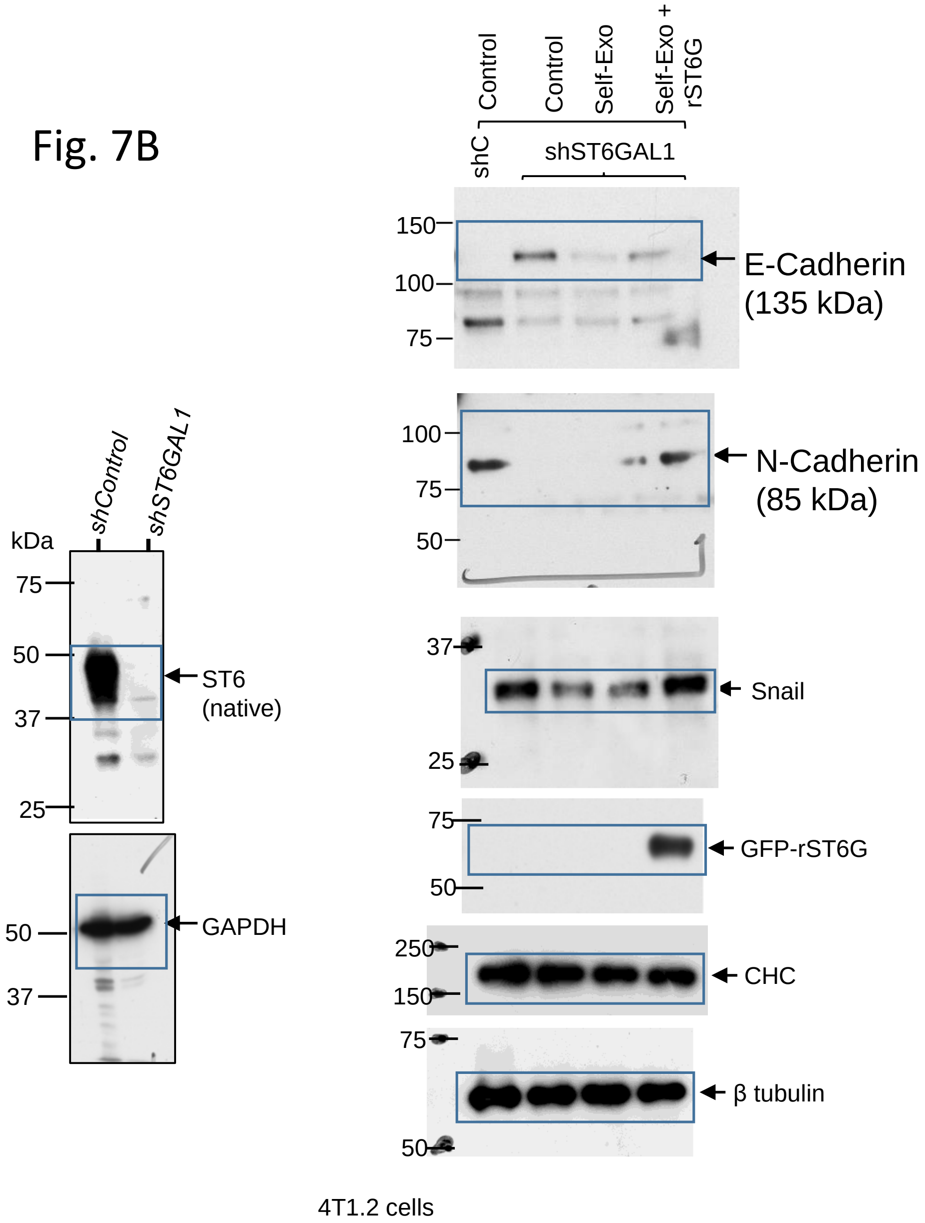

kDa
shControl
shST6GAL1
75
50
37
25
ST6 (native)
50
37
GAPDH
shC
Control
Control
Self-Exo
Self-Exo + rST6G
shST6GAL1
150
100
75
E-Cadherin (135 kDa)
100
75
50
N-Cadherin (85 kDa)
37
25
Snail
75
50
GFP-rST6G
250
150
CHC
75
50
β tubulin
4T1.2 cells

Fig. 7D

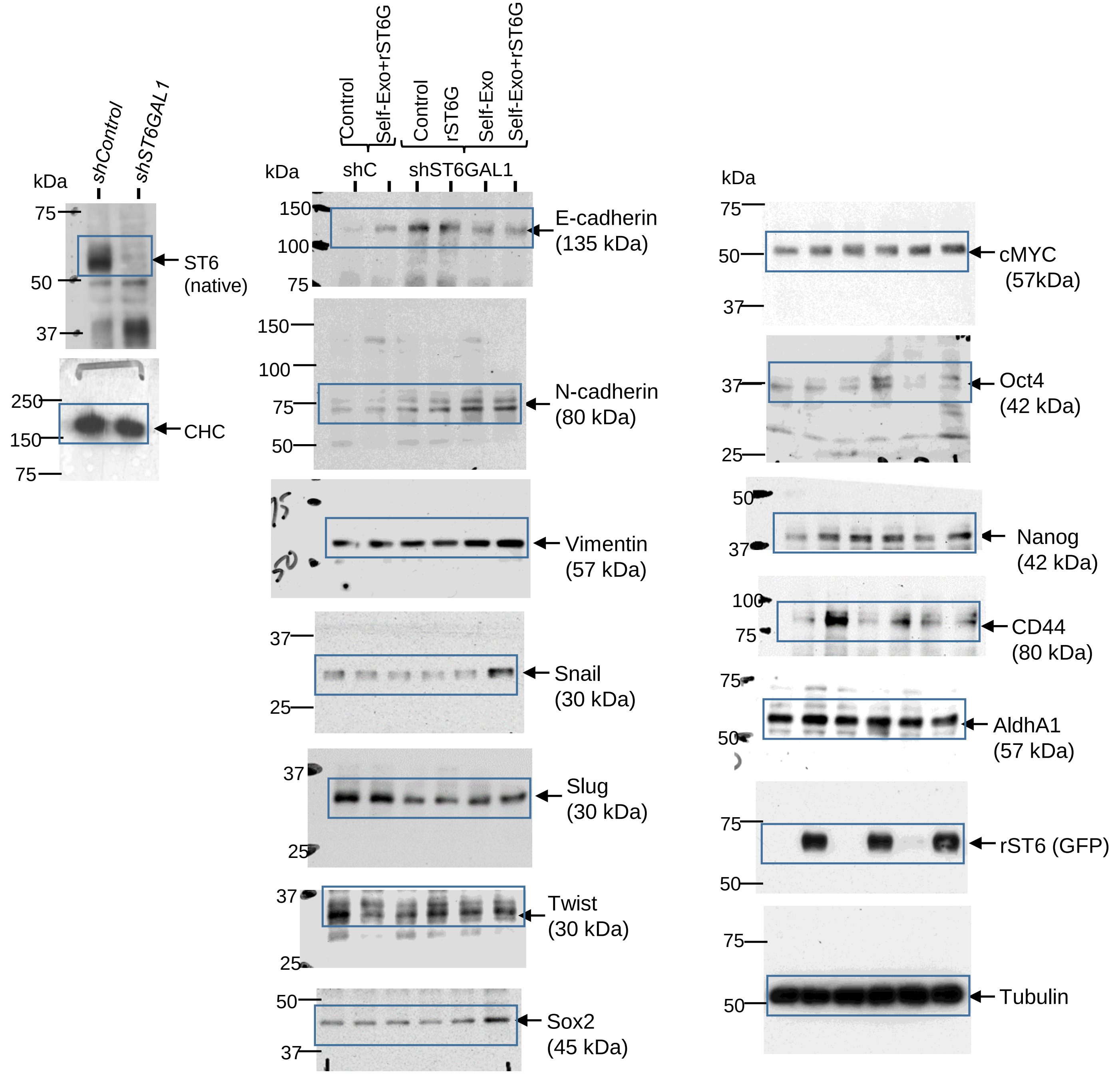

shControl
shST6GAL1
kDa
75
50
37
ST6 (native)
250
150
75
CHC
Control
Self-Exo+rST6G
Control
rST6G
Self-Exo
Self-Exo+rST6G
shC
shST6GAL1
kDa
150
100
75
E-cadherin (135 kDa)
150
100
75
50
N-cadherin (80 kDa)
Vimentin (57 kDa)
37
25
Snail (30 kDa)
37
25
Slug (30 kDa)
37
25
Twist (30 kDa)
50
37
Sox2 (45 kDa)
kDa
75
50
37
cMYC (57kDa)
37
25
Oct4 (42 kDa)
50
37
Nanog (42 kDa)
100
75
CD44 (80 kDa)
75
50
AldhA1 (57 kDa)
75
50
rST6 (GFP)
75
50
Tubulin

Well	Fluor	Target	Target	Sample	Cq	Cq					
**Original qPCR File for Fig 2A**					Ct values	Ct Values					N=3
				Samples	GAPDH	ST6GAL1	Delta Ct	2^(-dCT)x100	Ave.	S.D.	S.E.
**A01**	SYBR	GAPDH	ST6GAL1	MCF7	23.22	33.05	9.83	0.110	0.09	0.03	0.01
**A02**	SYBR	GAPDH	ST6GAL1	MCF7	23.32	33.93	10.61	0.064			
**A03**	SYBR	GAPDH	ST6GAL1	MCF7	23.57	33.44	9.87	0.107			
**A04**	SYBR	GAPDH	ST6GAL1	BT474	16.25	26.40	10.15	0.088	0.09	0.02	0.01
**A05**	SYBR	GAPDH	ST6GAL1	BT474	16.40	26.96	10.56	0.066			
**A06**	SYBR	GAPDH	ST6GAL1	BT474	16.30	26.17	9.87	0.107			
**A07**	SYBR	GAPDH	ST6GAL1	T47D	22.31	30.41	8.11	0.363	0.39	0.04	0.02
**A08**	SYBR	GAPDH	ST6GAL1	T47D	22.22	30.07	7.85	0.432			
**A09**	SYBR	GAPDH	ST6GAL1	T47D	22.10	30.19	8.09	0.366			
**A10**	SYBR	GAPDH	ST6GAL1	ZR75-1	22.10	31.39	9.29	0.160	0.17	0.01	0.01
**A11**	SYBR	GAPDH	ST6GAL1	ZR75-1	22.10	31.25	9.15	0.176			
**A12**	SYBR	GAPDH	ST6GAL1	ZR75-1	22.10	31.23	9.14	0.178			
**B01**	SYBR	GAPDH	ST6GAL1	BT549	21.54	30.09	8.55	0.266	0.31	0.07	0.04
**B02**	SYBR	GAPDH	ST6GAL1	BT549	21.54	30.02	8.48	0.280			
**B03**	SYBR	GAPDH	ST6GAL1	BT549	21.54	29.55	8.01	0.388			
**B04**	SYBR	GAPDH	ST6GAL1	MDA231	17.58	26.29	8.71	0.239	0.21	0.04	0.02
**B05**	SYBR	GAPDH	ST6GAL1	MDA231	17.58	26.82	9.24	0.165			
**B06**	SYBR	GAPDH	ST6GAL1	MDA231	17.58	26.28	8.70	0.240			
**B07**	SYBR	GAPDH	ST6GAL1	LM-2-4	19.44	29.01	9.57	0.132	0.12	0.01	0.01
**B08**	SYBR	GAPDH	ST6GAL1	LM-2-4	19.28	29.00	9.73	0.118			
**B09**	SYBR	GAPDH	ST6GAL1	LM-2-4	19.36	29.14	9.77	0.114			
**B10**	SYBR	GAPDH	ST6GAL1	231BR	18.18	27.13	8.95	0.202	0.27	0.07	0.04
**B11**	SYBR	GAPDH	ST6GAL1	231BR	18.85	27.03	8.18	0.345			
**B12**	SYBR	GAPDH	ST6GAL1	231BR	18.05	26.68	8.63	0.253			
**C01**	SYBR	GAPDH	ST6GAL1	4T1.	27.28	26.20	-1.08	210.865	238.09	48.65	28.09
**C02**	SYBR	GAPDH	ST6GAL1	4T1.	27.88	26.82	-1.06	209.140			
**C03**	SYBR	GAPDH	ST6GAL1	4T1.	27.64	26.09	-1.56	294.256			
**C04**	SYBR	GAPDH	ST6GAL1	4T1.2	28.44	29.47	1.03	48.846	40.97	7.40	4.27
**C05**	SYBR	GAPDH	ST6GAL1	4T1.2	27.75	29.30	1.55	34.159			
**C06**	SYBR	GAPDH	ST6GAL1	4T1.2	28.00	29.33	1.33	39.909			
**C07**	SYBR	GAPDH	ST6GAL1	E0771	26.49	31.05	4.55	4.260	3.76	0.66	0.38
**C08**	SYBR	GAPDH	ST6GAL1	E0771	26.80	31.44	4.64	4.003			
**C09**	SYBR	GAPDH	ST6GAL1	E0771	26.79	31.84	5.05	3.011			

Well	Fluor	Target	Target	Sample	Cq	Cq						Ave
**Original qPCR File for Fig 3A**					Ct values	Ct Values					N=3	dCt
				Samples	GAPDH	ST6GAL1	dCT	ddCT	2^(-ddCT)	Ave.	S.D.	
**A01**	SYBR	GAPDH	ST6GAL1	4T1.2 sh Cont	22.43	19.67	2.76	0.16	0.89	1.00	0.09	2.60
**A02**	SYBR	GAPDH	ST6GAL1	4T1.2 sh Cont	22.32	19.79	2.52	-0.08	1.05			
**A03**	SYBR	GAPDH	ST6GAL1	4T1.2 sh Cont	22.36	19.84	2.52	-0.08	1.05			
**A04**	SYBR	GAPDH	ST6GAL1	4T1.2 shST6	40.00	28.85	11.15	8.55	0.00	0.01	0.01	
**A05**	SYBR	GAPDH	ST6GAL1	4T1.2 shST6	40.00	28.69	11.31	8.71	0.00			
**A06**	SYBR	GAPDH	ST6GAL1	4T1.2 shST6	38.00	29.26	8.74	6.14	0.01			

**Original qPCR File for Fig 3D**										**N=3**	**Ave.**
**BT-549 cells**	Fluor	Samples	CT (ST6GAL1)		CT (GAPDH)	dCT	ddCT	2^(-ddCT)	Ave.	S.D.	dCT
**shControl**	SYBR	BT549-sh Cont	22.43		20.67	1.76	0.2	0.89	1.0011	0.0921	1.60
	SYBR	BT549-sh Cont	22.32		20.79	1.52	-0.1	1.05			
	SYBR	BT549-sh Cont	22.36		20.84	1.52	-0.1	1.05			
**shST6GAL-1 #1**	SYBR	BT549-shST6-1	28.00		20.85	7.15	5.5	0.02	0.0626	0.0778	
	SYBR	BT549-shST6-1	27.00		22.69	4.31	2.7	0.15			
	SYBR	BT549-shST6-1	31.00		23.26	7.74	6.1	0.01			
**shST6GAL-1 #2**	SYBR	BT549-shST6-2	29.00		20.69	8.31	6.7	0.01	0.0545	0.0848	
	SYBR	BT549-shST6-2	32.00		21.26	10.74	9.1	0.00			
	SYBR	BT549-shST6-2	26.00		21.69	4.31	2.7	0.15			

**Original qPCR File for Fig 7C**				**Ct**	**Ct**	**Delta**	**Delta-Delta**				**N=3**	**Delta Ct**
**Well**	**Fluor**	Target	Samples	ST6GAL1	GPDH	Ct	Ct	2^(-ddct)	AVE.	S.D.	S.E.	AVE.
**A01**	SYBR	ST6GAL1	T47D_1_cnt	24.64	14.69	9.95	(0.04)	1.03	1.00	0.05	0.03	9.99
**A02**	SYBR	ST6GAL1	T47D_1_cnt	24.57	14.62	9.94	(0.05)	1.03				
**A03**	SYBR	ST6GAL1	T47D_1_cnt	24.66	14.59	10.07	0.08	0.95				
**A04**	SYBR	ST6GAL1	T47D_2_cnt+	24.70	14.42	10.28	0.29	0.82	0.89	0.09	0.05	
**A05**	SYBR	ST6GAL1	T47D_2_cnt+	24.48	14.49	9.99	0.00	1.00				
**A06**	SYBR	ST6GAL1	T47D_2_cnt+	24.55	14.33	10.21	0.22	0.86				
**A07**	SYBR	ST6GAL1	T47D_3_Sh#1	26.25	14.45	11.80	1.81	0.29	0.24	0.10	0.06	
**A08**	SYBR	ST6GAL1	T47D_3_Sh#1	26.25	14.59	11.65	1.66	0.32				
**A09**	SYBR	ST6GAL1	T47D_3_Sh#1	26.11	13.16	12.95	2.96	0.13				
**A10**	SYBR	ST6GAL1	T47D_4+ rST6	26.13	14.30	11.83	1.84	0.28	0.27	0.02	0.01	
**A11**	SYBR	ST6GAL1	T47D_4+ rST6	26.17	14.34	11.83	1.84	0.28				
**A12**	SYBR	ST6GAL1	T47D_4+ rST6	26.31	14.27	12.04	2.05	0.24				
**B01**	SYBR	ST6GAL1	T47D_5+EXO	26.77	15.17	11.61	1.62	0.33	0.32	0.00	0.00	
**B02**	SYBR	ST6GAL1	T47D_5+EXO	26.70	15.10	11.60	1.61	0.33				
**B03**	SYBR	ST6GAL1	T47D_5+EXO	26.67	15.04	11.63	1.64	0.32				
**B04**	SYBR	ST6GAL1	T47D_6+EXO/rST6	27.16	15.69	11.48	1.49	0.36	0.31	0.04	0.02	
**B05**	SYBR	ST6GAL1	T47D_6+EXO/rST6	27.12	15.45	11.67	1.68	0.31				
**B06**	SYBR	ST6GAL1	T47D_6+EXO/rST6	27.23	15.37	11.86	1.87	0.27				

				**Ct**	**Ct**	**Delta**	**Delta-Delta**					**Delta Ct**

E-Cadherin gene				E-Cadherin	GPDH	Ct	Ct	2^(-ddct)	AVE.	S.D.	S.E.	AVE.
**A01**	SYBR	E-Cadherin	T47D_1_cnt	34.75	14.69	20.06	0.12	0.92	1.02	0.25	0.14	19.939
**A02**	SYBR	E-Cadherin	T47D_1_cnt	34.82	14.62	20.20	0.26	0.83				
**A03**	SYBR	E-Cadherin	T47D_1_cnt	34.15	14.59	19.56	(0.38)	1.30				
**A04**	SYBR	E-Cadherin	T47D_2_cnt+	35.81	14.42	21.39	1.45	0.37	0.43	0.06	0.03	
**A05**	SYBR	E-Cadherin	T47D_2_cnt+	35.56	14.49	21.07	1.14	0.46				
**A06**	SYBR	E-Cadherin	T47D_2_cnt+	35.34	14.33	21.01	1.07	0.48				
**A07**	SYBR	E-Cadherin	T47D_3_Sh#1	33.01	14.45	18.56	(1.37)	2.59	2.54	0.38	0.22	
**A08**	SYBR	E-Cadherin	T47D_3_Sh#1	33.00	14.59	18.41	(1.53)	2.89				
**A09**	SYBR	E-Cadherin	T47D_3_Sh#1	32.00	13.16	18.84	(1.09)	2.14				
**A10**	SYBR	E-Cadherin	T47D_4+ rST6	33.98	14.30	19.67	(0.27)	1.20	1.24	0.31	0.18	
**A11**	SYBR	E-Cadherin	T47D_4+ rST6	33.63	14.34	19.29	(0.65)	1.57				
**A12**	SYBR	E-Cadherin	T47D_4+ rST6	34.26	14.27	20.00	0.06	0.96				
**B01**	SYBR	E-Cadherin	T47D_5+EXO	35.09	15.17	19.92	(0.02)	1.01	1.24	0.21	0.12	
**B02**	SYBR	E-Cadherin	T47D_5+EXO	34.54	15.10	19.44	(0.49)	1.41				
**B03**	SYBR	E-Cadherin	T47D_5+EXO	34.59	15.04	19.55	(0.39)	1.31				
**B04**	SYBR	E-Cadherin	T47D_6+EXO/rST6	35.62	15.69	19.93	(0.00)	1.00	0.91	0.09	0.05	
**B05**	SYBR	E-Cadherin	T47D_6+EXO/rST6	35.67	15.45	20.22	0.28	0.82				
**B06**	SYBR	E-Cadherin	T47D_6+EXO/rST6	35.46	15.37	20.09	0.15	0.90				

**N-Cadherin gene**				**Ct**	**Ct**	**Delta**	**Delta-Delta**					**Delta Ct**
				N-Cadherin	GPDH	Ct	Ct	2^(-ddct)	AVE.	S.D.	S.E.	AVE.
**E01**	SYBR	N-Cadherin	T47D_1_cnt	34.07	14.69	19.37	0.065	0.956	1.059	0.45	0.26	19.31
**E02**	SYBR	N-Cadherin	T47D_1_cnt	34.51	14.62	19.88	0.573	0.672				
**E03**	SYBR	N-Cadherin	T47D_1_cnt	33.27	14.59	18.68	(0.630)	1.548				
**E04**	SYBR	N-Cadherin	T47D_2_cnt+	35.06	14.42	20.64	1.333	0.397	0.587	0.40	0.23	
**E05**	SYBR	N-Cadherin	T47D_2_cnt+	33.73	14.49	19.24	(0.066)	1.047				
**E06**	SYBR	N-Cadherin	T47D_2_cnt+	35.30	14.33	20.96	1.652	0.318				
**E07**	SYBR	N-Cadherin	T47D_3_Sh#1	34.00	14.45	19.56	0.249	0.841	0.793	0.32	0.18	
**E08**	SYBR	N-Cadherin	T47D_3_Sh#1	33.79	14.59	19.19	(0.116)	1.084				
**E09**	SYBR	N-Cadherin	T47D_3_Sh#1	33.60	13.16	20.45	1.136	0.455				
**E10**	SYBR	N-Cadherin	T47D_4+ rST6	30.21	14.30	15.90	(3.406)	10.598	12.932	4.49	2.59	
**E11**	SYBR	N-Cadherin	T47D_4+ rST6	29.47	14.34	15.13	(4.179)	18.111				
**E12**	SYBR	N-Cadherin	T47D_4+ rST6	30.25	14.27	15.98	(3.334)	10.086				
**F01**	SYBR	N-Cadherin	T47D_5+EXO	31.43	15.17	16.27	(3.042)	8.237	12.331	3.60	2.08	
**F02**	SYBR	N-Cadherin	T47D_5+EXO	30.62	15.10	15.52	(3.785)	13.785				
**F03**	SYBR	N-Cadherin	T47D_5+EXO	30.45	15.04	15.41	(3.904)	14.972				
**F04**	SYBR	N-Cadherin	T47D_6+EXO/rST6	30.02	15.69	14.34	(4.974)	31.439	27.267	5.82	3.36	
**F05**	SYBR	N-Cadherin	T47D_6+EXO/rST6	29.86	15.45	14.42	(4.894)	29.740				
**F06**	SYBR	N-Cadherin	T47D_6+EXO/rST6	30.32	15.37	14.94	(4.366)	20.622				

**Vimentin gene**				**Ct**	**Ct**	**Delta**	**Delta-Delta**					**Delta Ct**
				Vimentin	GPDH	Ct	Ct	2^(-ddct)	AVE.	S.D.	S.E.	AVE.
**E01**	SYBR	Vimentin	T47D_1_cnt	14.25	14.69	(0.44)	(0.809)	1.752	1.671	0.07	0.04	(0.37)
**E02**	SYBR	Vimentin	T47D_1_cnt	14.27	14.62	(0.35)	(0.721)	1.648				
**E03**	SYBR	Vimentin	T47D_1_cnt	14.27	14.59	(0.32)	(0.690)	1.614				
**E04**	SYBR	Vimentin	T47D_2_cnt+	14.03	14.42	(0.40)	(0.765)	1.700	2.313	0.86	0.50	

**E05**	SYBR	Vimentin	T47D_2_cnt+	13.90	14.49	(0.59)	(0.955)	1.939				
**E06**	SYBR	Vimentin	T47D_2_cnt+	12.98	14.33	(1.35)	(1.723)	3.300				
**E07**	SYBR	Vimentin	T47D_3_Sh#1	14.25	14.45	(0.20)	(0.566)	1.480	1.066	0.43	0.25	
**E08**	SYBR	Vimentin	T47D_3_Sh#1	14.84	14.59	0.24	(0.129)	1.093				
**E09**	SYBR	Vimentin	T47D_3_Sh#1	14.21	13.16	1.05	0.680	0.624				
**E10**	SYBR	Vimentin	T47D_4+ rST6	14.27	14.30	(0.03)	(0.400)	1.319	1.166	0.14	0.08	
**E11**	SYBR	Vimentin	T47D_4+ rST6	14.65	14.34	0.31	(0.055)	1.039				
**E12**	SYBR	Vimentin	T47D_4+ rST6	14.45	14.27	0.18	(0.187)	1.139				
**F01**	SYBR	Vimentin	T47D_5+EXO	14.92	15.17	(0.24)	(0.612)	1.529	1.401	0.12	0.07	
**F02**	SYBR	Vimentin	T47D_5+EXO	15.01	15.10	(0.09)	(0.455)	1.371				
**F03**	SYBR	Vimentin	T47D_5+EXO	15.03	15.04	(0.01)	(0.381)	1.302				
**F04**	SYBR	Vimentin	T47D_6+EXO/rST6	14.58	15.69	(1.11)	(1.481)	2.791	2.929	0.27	0.16	
**F05**	SYBR	Vimentin	T47D_6+EXO/rST6	14.12	15.45	(1.33)	(1.696)	3.240				
**F06**	SYBR	Vimentin	T47D_6+EXO/rST6	14.28	15.37	(1.09)	(1.462)	2.755				

**Snail gene**				**Ct**	**Ct**	**Delta**	**Delta-Delta**					**Delta Ct**
				Snail	GPDH	Ct	Ct	2^(-ddct)	AVE.	S.D.	S.E.	AVE.
**E01**	SYBR	Snail	T47D_1_cnt	25.11	14.69	10.42	(0.114)	1.082	1.002	0.07	0.04	10.53
**E02**	SYBR	Snail	T47D_1_cnt	25.20	14.62	10.57	0.043	0.971				
**E03**	SYBR	Snail	T47D_1_cnt	25.19	14.59	10.60	0.069	0.953				
**E04**	SYBR	Snail	T47D_2_cnt+	24.97	14.42	10.55	0.025	0.983	1.065	0.13	0.07	
**E05**	SYBR	Snail	T47D_2_cnt+	24.74	14.49	10.26	(0.274)	1.209				
**E06**	SYBR	Snail	T47D_2_cnt+	24.86	14.33	10.52	(0.005)	1.004				
**E07**	SYBR	Snail	T47D_3_Sh#1	24.92	14.45	10.47	(0.059)	1.042	0.878	0.38	0.22	
**E08**	SYBR	Snail	T47D_3_Sh#1	24.92	14.59	10.33	(0.203)	1.151				
**E09**	SYBR	Snail	T47D_3_Sh#1	24.87	13.16	11.71	1.185	0.440				
**E10**	SYBR	Snail	T47D_4+ rST6	24.36	14.30	10.05	(0.476)	1.391	1.239	0.13	0.08	
**E11**	SYBR	Snail	T47D_4+ rST6	24.66	14.34	10.32	(0.211)	1.158				
**E12**	SYBR	Snail	T47D_4+ rST6	24.58	14.27	10.31	(0.223)	1.167				
**F01**	SYBR	Snail	T47D_5+EXO	25.38	15.17	10.21	(0.318)	1.247	1.568	0.32	0.19	
**F02**	SYBR	Snail	T47D_5+EXO	24.71	15.10	9.61	(0.919)	1.891				
**F03**	SYBR	Snail	T47D_5+EXO	24.92	15.04	9.88	(0.649)	1.568				
**F04**	SYBR	Snail	T47D_6+EXO/rST6	24.04	15.69	8.36	(2.174)	4.513	3.958	0.48	0.28	
**F05**	SYBR	Snail	T47D_6+EXO/rST6	24.09	15.45	8.64	(1.889)	3.705				
**F06**	SYBR	Snail	T47D_6+EXO/rST6	24.03	15.37	8.66	(1.870)	3.656				

				**Ct**	**Ct**	**Delta**	**Delta-Delta**					**Delta Ct**
**SLUG gene**				SLUG	GPDH	Ct	Ct	2^(-ddct)	AVE.	S.D.	S.E.	AVE.
**A01**	SYBR	SLUG	T47D_1_cnt	23.06	14.69	8.37	(0.11)	1.08	1.004	0.07	0.04	8.476
**A02**	SYBR	SLUG	T47D_1_cnt	23.14	14.62	8.52	0.04	0.97				
**A03**	SYBR	SLUG	T47D_1_cnt	23.13	14.59	8.54	0.06	0.96				
**A04**	SYBR	SLUG	T47D_2_cnt+	23.16	14.42	8.74	0.26	0.83	0.846	0.11	0.06	
**A05**	SYBR	SLUG	T47D_2_cnt+	23.03	14.49	8.54	0.06	0.96				
**A06**	SYBR	SLUG	T47D_2_cnt+	23.24	14.33	8.90	0.42	0.75				
**A07**	SYBR	SLUG	T47D_3_Sh#1	23.53	14.45	9.08	0.60	0.66	0.558	0.20	0.12	
**A08**	SYBR	SLUG	T47D_3_Sh#1	23.61	14.59	9.01	0.53	0.69				
**A09**	SYBR	SLUG	T47D_3_Sh#1	23.27	13.16	10.11	1.63	0.32				

**A10**	SYBR	SLUG	T47D_4+ rST6	23.38	14.30	9.08	0.60	0.66	0.688	0.08	0.04	
**A11**	SYBR	SLUG	T47D_4+ rST6	23.49	14.34	9.15	0.67	0.63				
**A12**	SYBR	SLUG	T47D_4+ rST6	23.12	14.27	8.85	0.37	0.77				
**B01**	SYBR	SLUG	T47D_5+EXO	23.93	15.17	8.77	0.29	0.82	0.811	0.04	0.02	
**B02**	SYBR	SLUG	T47D_5+EXO	23.83	15.10	8.73	0.25	0.84				
**B03**	SYBR	SLUG	T47D_5+EXO	23.89	15.04	8.85	0.37	0.77				
**B04**	SYBR	SLUG	T47D_6+EXO/rST6	24.04	15.69	8.35	(0.13)	1.09	0.928	0.15	0.09	
**B05**	SYBR	SLUG	T47D_6+EXO/rST6	24.27	15.45	8.82	0.34	0.79				
**B06**	SYBR	SLUG	T47D_6+EXO/rST6	24.01	15.37	8.63	0.15	0.90				

				**Ct**	**Ct**	**Delta**	**Delta-Delta**					**Delta Ct**
**TWIST gene**				TWIST	GPDH	Ct	Ct	2^(-ddct)	AVE.	S.D.	S.E.	AVE.
**A01**	SYBR	TWIST	T47D_1_cnt	33.78	14.69	19.09	(0.41)	1.33	1.026	0.28	0.16	19.501
**A02**	SYBR	TWIST	T47D_1_cnt	34.51	14.62	19.89	0.39	0.77				
**A03**	SYBR	TWIST	T47D_1_cnt	34.11	14.59	19.52	0.02	0.98				
**A04**	SYBR	TWIST	T47D_2_cnt+	31.35	14.42	16.93	(2.57)	5.93	6.250	0.44	0.25	
**A05**	SYBR	TWIST	T47D_2_cnt+	31.39	14.49	16.90	(2.60)	6.07				
**A06**	SYBR	TWIST	T47D_2_cnt+	31.08	14.33	16.75	(2.76)	6.75				
**A07**	SYBR	TWIST	T47D_3_Sh#1	33.17	14.45	18.72	(0.78)	1.71	1.408	0.30	0.17	
**A08**	SYBR	TWIST	T47D_3_Sh#1	33.93	14.59	19.34	(0.16)	1.12				
**A09**	SYBR	TWIST	T47D_3_Sh#1	32.18	13.16	19.03	(0.47)	1.39				
**A10**	SYBR	TWIST	T47D_4+ rST6	30.75	14.30	16.44	(3.06)	8.33	10.433	4.36	2.52	
**A11**	SYBR	TWIST	T47D_4+ rST6	30.93	14.34	16.59	(2.91)	7.52				
**A12**	SYBR	TWIST	T47D_4+ rST6	29.82	14.27	15.55	(3.95)	15.45				
**B01**	SYBR	TWIST	T47D_5+EXO	29.99	15.17	14.83	(4.68)	25.56	24.243	2.92	1.68	
**B02**	SYBR	TWIST	T47D_5+EXO	29.89	15.10	14.79	(4.72)	26.27				
**B03**	SYBR	TWIST	T47D_5+EXO	30.16	15.04	15.12	(4.39)	20.90				
**B04**	SYBR	TWIST	T47D_6+EXO/rST6	29.59	15.69	13.90	(5.60)	48.58	43.659	4.30	2.48	
**B05**	SYBR	TWIST	T47D_6+EXO/rST6	29.56	15.45	14.12	(5.39)	41.81				
**B06**	SYBR	TWIST	T47D_6+EXO/rST6	29.53	15.37	14.16	(5.34)	40.59				

				**Ct**	**Ct**	**Delta**	**Delta-Delta**					**Delta Ct**
**SOX2 gene**				SOX2	GPDH	Ct	Ct	2^(-ddct)	AVE.	S.D.	S.E.	AVE.
**A01**	SYBR	SOX2	T47D_1_cnt	31.19	14.69	16.49	0.04	0.98	1.001	0.04	0.02	16.458
**A02**	SYBR	SOX2	T47D_1_cnt	31.02	14.62	16.39	(0.07)	1.05				
**A03**	SYBR	SOX2	T47D_1_cnt	31.08	14.59	16.49	0.03	0.98				
**A04**	SYBR	SOX2	T47D_2_cnt+	30.41	14.42	15.99	(0.47)	1.38	1.188	0.24	0.14	
**A05**	SYBR	SOX2	T47D_2_cnt+	30.61	14.49	16.12	(0.34)	1.27				
**A06**	SYBR	SOX2	T47D_2_cnt+	30.92	14.33	16.59	0.13	0.91				
**A07**	SYBR	SOX2	T47D_3_Sh#1	30.99	14.45	16.54	0.09	0.94	0.837	0.45	0.26	
**A08**	SYBR	SOX2	T47D_3_Sh#1	30.76	14.59	16.16	(0.30)	1.23				
**A09**	SYBR	SOX2	T47D_3_Sh#1	31.17	13.16	18.01	1.55	0.34				
**A10**	SYBR	SOX2	T47D_4+ rST6	30.14	14.30	15.84	(0.62)	1.53	1.213	0.38	0.22	
**A11**	SYBR	SOX2	T47D_4+ rST6	30.41	14.34	16.07	(0.38)	1.30				
**A12**	SYBR	SOX2	T47D_4+ rST6	31.05	14.27	16.78	0.32	0.80				
**B01**	SYBR	SOX2	T47D_5+EXO	29.45	15.17	14.28	(2.18)	4.53	4.712	0.79	0.45	
**B02**	SYBR	SOX2	T47D_5+EXO	29.55	15.10	14.45	(2.01)	4.03				

**B03**	SYBR	SOX2	T47D_5+EXO	29.02	15.04	13.98	(2.48)	5.58				
**B04**	SYBR	SOX2	T47D_6+EXO/rST 6	29.85	15.69	14.16	(2.30)	4.92	4.505	0.44	0.25	
**B05**	SYBR	SOX2	T47D_6+EXO/rST 6	29.72	15.45	14.27	(2.18)	4.54				
**B06**	SYBR	SOX2	T47D_6+EXO/rST 6	29.81	15.37	14.44	(2.02)	4.05				

				**Ct**	**Ct**	**Delta**	**Delta-Delta**					**Delta Ct**
**MYC gene**				MYC	GPDH	Ct	Ct	2^(-ddct)	AVE.	S.D.	S.E.	AVE.
**A01**	SYBR	MYC	T47D_1_cnt	20.09	14.69	5.40	0.32	0.80	1.020	0.25	0.15	5.080
**A02**	SYBR	MYC	T47D_1_cnt	19.76	14.62	5.14	0.06	0.96				
**A03**	SYBR	MYC	T47D_1_cnt	19.30	14.59	4.71	(0.37)	1.30				
**A04**	SYBR	MYC	T47D_2_cnt+	19.44	14.42	5.02	(0.06)	1.04	1.091	0.04	0.03	
**A05**	SYBR	MYC	T47D_2_cnt+	19.42	14.49	4.94	(0.14)	1.11				
**A06**	SYBR	MYC	T47D_2_cnt+	19.25	14.33	4.91	(0.17)	1.12				
**A07**	SYBR	MYC	T47D_3_Sh#1	19.54	14.45	5.09	0.01	0.99	0.885	0.36	0.21	
**A08**	SYBR	MYC	T47D_3_Sh#1	19.44	14.59	4.84	(0.24)	1.18				
**A09**	SYBR	MYC	T47D_3_Sh#1	19.28	13.16	6.12	1.04	0.49				
**A10**	SYBR	MYC	T47D_4+ rST6	19.56	14.30	5.25	0.17	0.89	0.796	0.08	0.05	
**A11**	SYBR	MYC	T47D_4+ rST6	19.78	14.34	5.44	0.36	0.78				
**A12**	SYBR	MYC	T47D_4+ rST6	19.82	14.27	5.55	0.47	0.72				
**B01**	SYBR	MYC	T47D_5+EXO	19.35	15.17	4.18	(0.90)	1.86	1.824	0.25	0.14	
**B02**	SYBR	MYC	T47D_5+EXO	19.14	15.10	4.04	(1.04)	2.05				
**B03**	SYBR	MYC	T47D_5+EXO	19.48	15.04	4.44	(0.64)	1.56				
**B04**	SYBR	MYC	T47D_6+EXO/rST6	19.33	15.69	3.64	(1.44)	2.71	2.386	0.28	0.16	
**B05**	SYBR	MYC	T47D_6+EXO/rST6	19.36	15.45	3.91	(1.17)	2.24				
**B06**	SYBR	MYC	T47D_6+EXO/rST6	19.31	15.37	3.94	(1.14)	2.21				
				Ct	Ct	Delta	Delta-Delta					Delta Ct
**Oct4 gene**				(Oct4)	GPDH	Ct	Ct	2^(-ddct)	AVE.	S.D.	S.E.	AVE.
**A01**	SYBR	OCT4	T47D_1_cnt	23.16	14.69	8.47	(0.08)	1.06	1.001	0.05	0.03	8.545
**A02**	SYBR	OCT4	T47D_1_cnt	23.19	14.62	8.57	0.02	0.99				
**A03**	SYBR	OCT4	T47D_1_cnt	23.19	14.59	8.60	0.06	0.96				
**A04**	SYBR	OCT4	T47D_2_cnt+	23.11	14.42	8.69	0.15	0.90	0.893	0.06	0.04	
**A05**	SYBR	OCT4	T47D_2_cnt+	23.11	14.49	8.62	0.08	0.95				
**A06**	SYBR	OCT4	T47D_2_cnt+	23.15	14.33	8.82	0.27	0.83				
**A07**	SYBR	OCT4	T47D_3_Sh#1	23.58	14.45	9.13	0.59	0.67	0.658	0.28	0.16	
**A08**	SYBR	OCT4	T47D_3_Sh#1	23.24	14.59	8.65	0.10	0.93				
**A09**	SYBR	OCT4	T47D_3_Sh#1	23.11	13.16	9.96	1.41	0.38				
**A10**	SYBR	OCT4	T47D_4+ rST6	23.11	14.30	8.80	0.26	0.84	0.853	0.07	0.04	
**A11**	SYBR	OCT4	T47D_4+ rST6	23.22	14.34	8.88	0.34	0.79				
**A12**	SYBR	OCT4	T47D_4+ rST6	22.92	14.27	8.65	0.10	0.93				
**B01**	SYBR	OCT4	T47D_5+EXO	23.39	15.17	8.22	(0.32)	1.25	1.066	0.17	0.10	
**B02**	SYBR	OCT4	T47D_5+EXO	23.59	15.10	8.49	(0.05)	1.04				
**B03**	SYBR	OCT4	T47D_5+EXO	23.72	15.04	8.68	0.14	0.91				
**B04**	SYBR	OCT4	T47D_6+EXO/rST6	23.68	15.69	7.99	(0.55)	1.47	1.238	0.20	0.11	
**B05**	SYBR	OCT4	T47D_6+EXO/rST6	23.83	15.45	8.38	(0.16)	1.12				
**B06**	SYBR	OCT4	T47D_6+EXO/rST6	23.74	15.37	8.37	(0.18)	1.13				

				**Ct**	**Ct**	**Delta**	**Delta-Delta**					**Delta Ct**

Nanog gene				Nanog	GPDH	Ct	Ct	2^(-ddct)	AVE.	S.D.	S.E.	AVE.
**A01**	SYBR	Nanog	T47D_1_cnt	27.39	14.69	12.70	0.21	0.86	1.005	0.12	0.07	12.484
**A02**	SYBR	Nanog	T47D_1_cnt	26.99	14.62	12.36	(0.12)	1.09				
**A03**	SYBR	Nanog	T47D_1_cnt	26.98	14.59	12.39	(0.09)	1.07				
**A04**	SYBR	Nanog	T47D_2_cnt+	26.25	14.42	11.83	(0.66)	1.58	1.292	0.27	0.15	
**A05**	SYBR	Nanog	T47D_2_cnt+	26.65	14.49	12.16	(0.32)	1.25				
**A06**	SYBR	Nanog	T47D_2_cnt+	26.75	14.33	12.42	(0.07)	1.05				
**A07**	SYBR	Nanog	T47D_3_Sh#1	28.94	14.45	14.49	2.01	0.25	0.224	0.11	0.06	
**A08**	SYBR	Nanog	T47D_3_Sh#1	28.73	14.59	14.13	1.65	0.32				
**A09**	SYBR	Nanog	T47D_3_Sh#1	28.91	13.16	15.75	3.27	0.10				
**A10**	SYBR	Nanog	T47D_4+ rST6	26.55	14.30	12.25	(0.24)	1.18	1.173	0.05	0.03	
**A11**	SYBR	Nanog	T47D_4+ rST6	26.67	14.34	12.33	(0.16)	1.11				
**A12**	SYBR	Nanog	T47D_4+ rST6	26.46	14.27	12.19	(0.29)	1.22				
**B01**	SYBR	Nanog	T47D_5+EXO	28.41	15.17	13.24	0.76	0.59	0.567	0.02	0.01	
**B02**	SYBR	Nanog	T47D_5+EXO	28.46	15.10	13.36	0.87	0.55				
**B03**	SYBR	Nanog	T47D_5+EXO	28.35	15.04	13.31	0.83	0.56				
**B04**	SYBR	Nanog	T47D_6+EXO/rST6	26.02	15.69	10.33	(2.15)	4.45	4.186	0.34	0.20	
**B05**	SYBR	Nanog	T47D_6+EXO/rST6	26.00	15.45	10.56	(1.93)	3.80				
**B06**	SYBR	Nanog	T47D_6+EXO/rST6	25.75	15.37	10.38	(2.11)	4.31				

**CD44 gene**				**Ct**	**Ct**	**Delta**	**Delta-Delta**					**Delta Ct**
				CD44	GPDH	Ct	Ct	2^(-ddct)	AVE.	S.D.	S.E.	AVE.
**E01**	SYBR	CD44	T47D_1_cnt	17.13	14.69	2.44	0.227	0.855	1.025	0.29	0.17	2.21
**E02**	SYBR	CD44	T47D_1_cnt	17.06	14.62	2.43	0.222	0.857				
**E03**	SYBR	CD44	T47D_1_cnt	16.35	14.59	1.76	(0.446)	1.362				
**E04**	SYBR	CD44	T47D_2_cnt+	16.92	14.42	2.50	0.285	0.821	0.848	0.15	0.09	
**E05**	SYBR	CD44	T47D_2_cnt+	16.68	14.49	2.19	(0.017)	1.012				
**E06**	SYBR	CD44	T47D_2_cnt+	17.04	14.33	2.70	0.492	0.711				
**E07**	SYBR	CD44	T47D_3_Sh#1	16.36	14.45	1.91	(0.298)	1.230	0.961	0.42	0.24	
**E08**	SYBR	CD44	T47D_3_Sh#1	16.57	14.59	1.98	(0.235)	1.177				
**E09**	SYBR	CD44	T47D_3_Sh#1	16.44	13.16	3.28	1.072	0.476				
**E10**	SYBR	CD44	T47D_4+ rST6	16.65	14.30	2.35	0.142	0.906	1.042	0.17	0.10	
**E11**	SYBR	CD44	T47D_4+ rST6	16.56	14.34	2.22	0.012	0.992				
**E12**	SYBR	CD44	T47D_4+ rST6	16.18	14.27	1.92	(0.295)	1.227				
**F01**	SYBR	CD44	T47D_5+EXO	16.64	15.17	1.47	(0.736)	1.665	1.414	0.28	0.16	
**F02**	SYBR	CD44	T47D_5+EXO	17.16	15.10	2.06	(0.155)	1.113				
**F03**	SYBR	CD44	T47D_5+EXO	16.70	15.04	1.66	(0.548)	1.462				
**F04**	SYBR	CD44	T47D_6+EXO/rST6	16.00	15.69	0.31	(1.896)	3.721	3.265	0.40	0.23	
**F05**	SYBR	CD44	T47D_6+EXO/rST6	16.02	15.45	0.57	(1.637)	3.109				
**F06**	SYBR	CD44	T47D_6+EXO/rST6	16.02	15.37	0.64	(1.568)	2.964				

				**Ct**	**Ct**	**Delta**	**Delta-Delta**					**Delta Ct**
**AldH1 gene**				AldH1	GPDH	Ct	Ct	2^(-ddct)	AVE.	S.D.	S.E.	AVE.
**A01**	SYBR	AldH1	T47D_1_cnt	30.48	14.69	15.79	(0.22)	1.17	1.017	0.23	0.13	16.014
**A02**	SYBR	AldH1	T47D_1_cnt	31.05	14.62	16.43	0.42	0.75				
**A03**	SYBR	AldH1	T47D_1_cnt	30.42	14.59	15.83	(0.18)	1.14				
**A04**	SYBR	AldH1	T47D_2_cnt+	29.99	14.42	15.57	(0.44)	1.35	1.421	0.10	0.06	

**A05**	SYBR	AldH1	T47D_2_cnt+	30.03	14.49	15.55	(0.46)	1.38				
**A06**	SYBR	AldH1	T47D_2_cnt+	29.73	14.33	15.40	(0.61)	1.53				
**A07**	SYBR	AldH1	T47D_3_Sh#1	31.05	14.45	16.60	0.59	0.66	0.578	0.28	0.16	
**A08**	SYBR	AldH1	T47D_3_Sh#1	30.93	14.59	16.33	0.32	0.80				
**A09**	SYBR	AldH1	T47D_3_Sh#1	31.06	13.16	17.90	1.89	0.27				
**A10**	SYBR	AldH1	T47D_4+ rST6	30.99	14.30	16.69	0.68	0.63	0.779	0.28	0.16	
**A11**	SYBR	AldH1	T47D_4+ rST6	30.21	14.34	15.87	(0.14)	1.10				
**A12**	SYBR	AldH1	T47D_4+ rST6	31.00	14.27	16.73	0.72	0.61				
**B01**	SYBR	AldH1	T47D_5+EXO	30.40	15.17	15.23	(0.78)	1.72	1.784	0.21	0.12	
**B02**	SYBR	AldH1	T47D_5+EXO	30.41	15.10	15.31	(0.70)	1.62				
**B03**	SYBR	AldH1	T47D_5+EXO	30.04	15.04	15.00	(1.01)	2.01				
**B04**	SYBR	AldH1	T47D_6+EXO/rST6	30.41	15.69	14.72	(1.29)	2.45	2.268	0.22	0.13	
**B05**	SYBR	AldH1	T47D_6+EXO/rST6	30.23	15.45	14.78	(1.23)	2.34				
**B06**	SYBR	AldH1	T47D_6+EXO/rST6	30.37	15.37	15.00	(1.01)	2.02				
